# Social interactions impact on the dopaminergic system and drive individuality

**DOI:** 10.1038/s41467-018-05526-5

**Published:** 2018-08-06

**Authors:** N. Torquet, F. Marti, C. Campart, S. Tolu, C. Nguyen, V. Oberto, M. Benallaoua, J. Naudé, S. Didienne, N. Debray, S. Jezequel, L. Le Gouestre, B. Hannesse, J. Mariani, A. Mourot, P. Faure

**Affiliations:** 1 Sorbonne Université, UPMC Univ Paris 06, INSERM, CNRS, Neuroscience Paris Seine - Institut de Biologie Paris Seine (NPS - IBPS), 75005 Paris, France; 2 Sorbonne Université, UPMC Univ Paris 06, INSERM, CNRS, Biological Adaptation and Ageing - Institut de Biologie Paris Seine (B2A - IBPS), 75005 Paris, France; 3 APHP Hôpital Charles Foix, DHU FAST, Institut de la Longévité, Ivry-Sur-Seine, France; 4 Sorbonne Université, UPMC Univ Paris 06, INSERM, CNRS UMS, 28 Phénotypage du Petit Animal, 75005 Paris, France

## Abstract

Individuality is a striking feature of animal behavior. Individual animals differ in traits and preferences which shape their interactions and their prospects for survival. However, the mechanisms underlying behavioral individuation are poorly understood and are generally considered to be genetic-based. Here, we devised a large environment, Souris City, in which mice live continuously in large groups. We observed the emergence of individual differences in social behavior, activity levels, and cognitive traits, even though the animals had low genetic diversity (inbred C57BL/6J strain). We further show that the phenotypic divergence in individual behaviors was mirrored by developing differences in midbrain dopamine neuron firing properties. Strikingly, modifying the social environment resulted in a fast re-adaptation of both the animal’s traits and its dopamine firing pattern. Individuality can rapidly change upon social challenges, and does not just depend on the genetic status or the accumulation of small differences throughout development.

## Abstract (web summary)

Individual animals differ in behavioral traits, but the mechanisms underlying individuation are unclear. Here, the authors show that mice living in a ‘city’ develop individual behavior differences, associated with changes in dopamine cell firing, that can be reversed on moving them to a different social environment.

## Introduction

Individuality refers to differences that remain stable over time and contexts for a series of behavioral traits expressed among individuals of the same species^1–5^. Individuality is a ubiquitous feature of animal populations^6^. Evidence for phenotypic variability lead to extensive research on its adaptive significance and its ecological or evolutionary consequences^1,5,7–10^. The proximal mechanisms underlying phenotypic variability have been understudied^4^, yet they could provide important information on how animals differ in their choices, stress responses, or susceptibilities to diseases.

The emergence of individuality has been linked to genetic and environmental interactions^6,11^. Experiments with groups of near-clonal mice reared in a large and controlled environment have demonstrated behavioral divergence^12,13^, which may emerge from the magnification of small initial differences in the epigenetic status or micro-environment of the animal^11^. In this perspective, the combination of individual history and initial differences would form a unique path for each individual and may explain the phenotypic variability observed at the population level. Social relationships are part of the history of individuals, and likely have important roles in shaping individuality. Notably, social-stress studies identified susceptible and resilient animals^14,15^, while social hierarchy analyses revealed that dominant animals are seemingly less sensitive to the effects of drugs than subordinates^16^, but more susceptible to develop depression-like behavior^17^. Normal or pathological social relationships can thus greatly modify individual behaviors in mice. However, the role of social relationships in the emergence of phenotypic variability is poorly understood. Interactions within a group were proposed to result in social specialization^3^, but whether the composition of a social group can affect non-social behavioral traits (like exploratory behavior for example) and the underlying neuronal processes remain to be determined.

Here, we questioned the role of social relationships in the emergence of individuality. For that purpose, we developed an experimental setup that combines an environment where animals live together with a modular testing platform, where animals are tested individually. In this environment, mice have individual access to specific feeding-related tasks while their social, circadian, and cognitive behaviors are monitored continuously and for long periods of time using multiple sensors. This setup enables the translation of activity and cognitive assessments into a definition of individual traits and allows to confirm the emergence of individuals with stable behavioral differences within a group of mice. Furthermore, we found a correlation between the traits of an animal and its neuronal activity at the level of the decision-making dopamine (DA) system. Finally, manipulating the social environment is sufficient to modify both animal traits and the activity of its DA cells. Altogether these data indicate that, in isogenic mice and for a conserved environment, social relationships impact development of individuality, possibly by regulating the activity of the DAergic system.

## Results

### Automatic analysis of behavior in a naturalistic environment

**Fig. 1:**
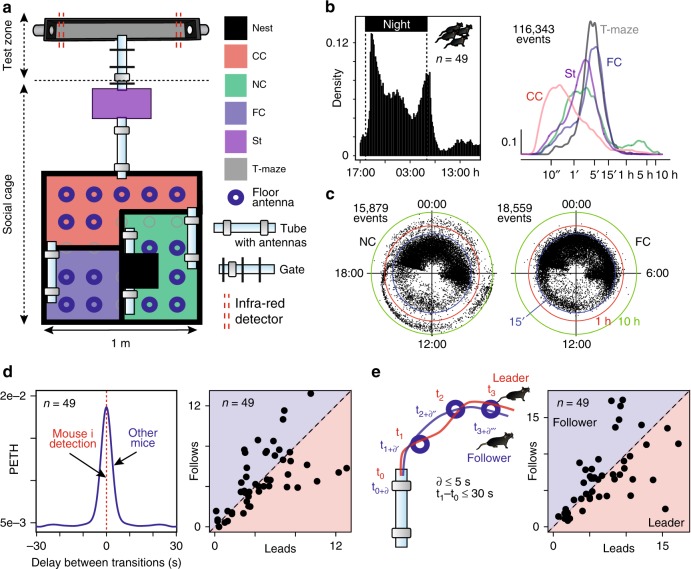
The Souris City environment. **a** Souris City setup with connectable sub-compartments, gates, and antennas. The setup is divided in two main parts: a social cage and a test zone. The social cage is divided in four sub-compartments: NC, which contains a nest, FC where mice have free and uncontrolled access to food, CC, and a stair (St) to get access to the gate (Supplementary Fig. 1). NC, FC, and CC are located in a 1 m ×1 m square, on which St is connected by a tube. Mice are tagged with RFID chips and detected by floor or tubes RFID antennas. A gate separates the test zone (here a T-maze) from the social cage. Two infrared beams (red dashed line) are used to detect mice in the T-maze. **b** (Left) Histogram of all the detection events from tubes (10 min time bins). (Right) Distribution of the time spent in each sub-compartment (log-scale, bandwidth = 0.1). **c** Circular plots showing the starting time (on a 24 h dial) and duration (log distance of the point to the center) of each visit (a dot) for NC and FC. Three circles indicate the 15′ (blue), 1 h (red), and 10 h (green) limits. **d** Analysis of social behavior: (Left) Peri-event time histogram (PETH density, bandwidth = 2 s) of transitions for distinct mice to the same sub-compartment (all sub-compartments pooled), indicating successive transitions within a 10 s window. (Right) Follower and leader mice, based on the ratio between the number of leads over the number of follows from sub-compartment transition episodes in a time window of 5 s. *n* = 49 mice from five experiments. **e** Chasing episodes are defined by concomitant detections of the same two mice on at least two consecutive antennas. (Right) Follower and leader mice, based on the ratio between the number of leads over the number of follows. *n* = 49 mice from five experiments. **d**, **e** Data were normalized with the duration of the session

Social life in natural environments and its consequences on the development of individuality cannot be easily addressed in standardized behavioral laboratory tests. Advances in automatic behavior analysis opens up new opportunities for in-depth phenotyping^18–20^ and for studying individuation in the laboratory^12^. An essential benefit of automation is the ability to conduct experiments on timescales that are orders of magnitude longer than traditional experiments (from minutes in classical assays to months of observation in automated systems). To test whether the social environment modifies individual traits, we first developed a complex and automatized environment, called Souris City, where male mice live in a group (10 to 20) for extended periods of time (2–3 months) while performing cognitive tests. Souris City is composed of a large environment (Social cage) connected to a test zone where individual animals, isolated from their conspecifics, performed a test (here a choice task in a T-maze to obtain water, Fig. 1a and Supplementary Fig. 1). Animals were RFID-tagged and detected by antennas (Supplementary Fig. 1). These detections were overall highly reliable (see limits in Supplementary Fig. 2), leading to an unambiguous global representation of mouse distribution within the different sub-compartments of Souris City: the nest sub-compartment (NC), food sub-compartment (FC), central sub-compartment (CC), stairs (St), and T-maze (Fig. 1a). The circadian rhythm of the group emerged from pooled (*n* = 49 mice; five experiments) activity measurement (Fig. 1b, left). The time spent by mice in a given sub-compartment generally varied between 1 and 30 min (Fig. 1b, right), with the shortest visits in CC, corresponding to transition episodes. Conversely, very long stays (several hours) were found in NC, especially during the light time (Fig. 1c, see also Supplementary Fig. 3) and were associated with sleep episodes. These variables described the general activity of the animals and were used to construct more complex representations, such as the entropy of their distribution (see Methods). Variables describing group behavior could also be extracted, mainly using indicators that translate the simultaneous presence of a group of animals in a given sub-compartment (e.g., CC, NC, FC…). Finally, a high rate of successive distinct RFID detections on tube-antennas within short time intervals were observed (Fig. 1d), indicating group dynamics and social events, i.e. two mice sequentially transitioning from one sub-compartment to another. Similarly, consecutive detections along different tube-antennas or floor-antennas (Fig. 1e, left) may indicate a follower tracking or chasing a leader (Fig. 1e, right).

### The emergence of individual profiles in Souris City

**Fig. 2:**
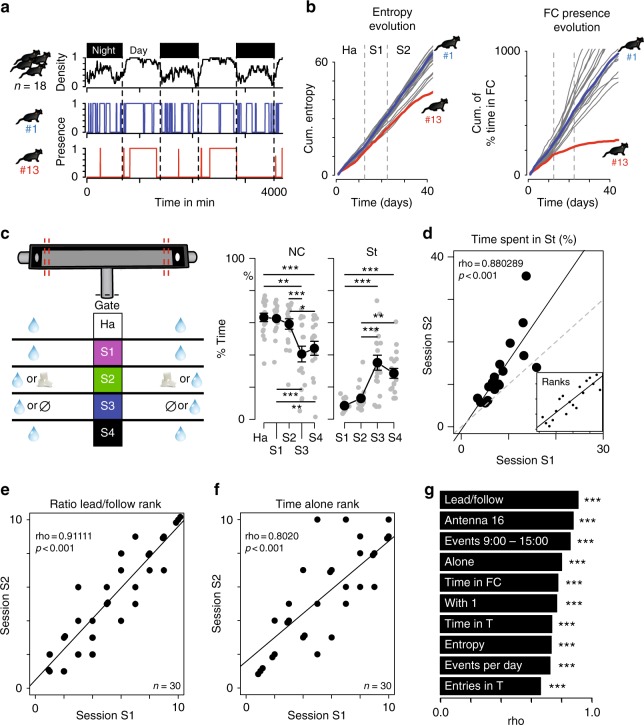
Consistency of behavior across situations. **a** Example of atypical behaviors. Top: density of mice in NC. Below: Presence ( = 1) of two mice (#1 and #13) in NC. **b** Cumulative distributions of entropy (left) and of the proportion of time spent in FC (right). **c** (Left) Diagram representing the five sessions. Variation across sessions (mean ± sem) of (middle) the proportion of time spent in NC (*n* = 18, F(4,68) = 22.69, *p* < 0.001; and post-hoc test) and (right) in St (*n* = 18, *F*(3,51) = 30.52, *p* < 0.001; and post-hoc test). **d** Correlation between proportion of time spent in St for individual mice in session 1 (S1) against session 2 (S2) (Spearman correlation coefficient, *n* = 18, fitted line = solid line, identity line = dotted line). Inset displays ranks instead of values with the correlation line. **e**, **f** Same as **d** inset for **e** the rank based on the ratio of leading over following and **f** the rank based on the proportion of time spent alone. **g** Rank correlations (rho) for two consecutive periods, for ten individual and social behaviors: lead/follow (see Fig. 1e); Antenna 16: number of detections on antenna #16 (floor antenna in the top right of the social cage, in CC); Events 9:0-15:0: number of events between 9:00 and 15:00; Alone: time spent in a sub-compartment with no other mice; Time in FC; With 1: time spent in a sub-compartment with one other mouse; Time in T: time spent in the T-maze; Entropy (see Methods); Events per day: number of tube antenna detections per day; Entries in T: number of entries in the T-maze. All these data were corrected by the duration of the session. For **e**–**g**, *n* = 30 mice from three independent experiments. ***p < 0.001,**p < 0.01,*p < 0.05

Long-term exposition to complex and large social environments was shown to elicit a magnification of individual differences in groups of genetically identical mice^12^. In agreement with this previous report, we observed in Souris City (i) a large variety of profiles (i.e., a set of behavioral measures such as active or social mice), including atypical ones (i.e., mice with singular profiles characterized by large divergence from the group, Fig. 2a, b), and (ii) the progressive divergence of individual measures linked to space occupancy, such as the entropy of animal distribution (Fig. 2b, left), the time spent in a given sub-compartment (Fig. 2b, right) or the time spent alone (Supplementary Fig. 4A). These observations suggest a marked consistency in individual behaviors over time, which is what defines the notion of individuality. To further substantiate the emergence of individuality, we quantified in the same experiment (*n* = 18 mice) behavioral correlations upon context variations. We performed five sessions for this experiment (Fig. 2c, left) in which both the rules to access drink dispensers and the drinking solutions were modified. Indeed, access to the T-maze, and thus to the drink dispensers, can be controlled by a gate allowing the selective entry of one mouse at a time (see Supplementary Fig. 1). During the habituation period (Ha), mice explored Souris City and had free access to water (gate always open). Then, access was gate-restricted and the reward associated with drink delivery was modified along four sessions: water on both sides (session S1), water or sucrose 5% (S2), water or nothing (S3), and finally back to water on both sides (S4). Overall, such manipulations altered the territorial organization in the social cage with variations of space occupancy in the nest and stair sub-compartments throughout the different sessions (Fig. 2c middle and right, Supplementary Fig. 4B). The modification of average behaviors across contexts contrasted with the stability of individual behaviors. For instance, animals spending less time than their conspecifics in the stair sub-compartment in S1 correspondingly spent less time in this sub-compartment in S2 (Fig. 2d), showing a behavioral consistency throughout the experiment for any given animal. In order to generalize these observations, we then realized three independent experiments (*n* = 10 animals in each) with three sessions (Ha, S1, and S2) in each. We then quantified behavioral consistency by examining the stability of ranking throughout S1 and S2 for a series of variables. Similarly, a large set of behaviors showed strong stability throughout the sessions, such as the animal inclination to lead or follow in chasing episodes (Fig. 2e), the proportion of time spent alone (Fig. 2f), as well as for additional social and non-social traits (Fig. 2g, Supplementary Fig. 4C). Overall, our results establish that mice developed individual profiles in this large environment, i.e., they maintained unique and coherent behavioral trajectories throughout time and situations.

### Different strategies of decision-making outside the group

**Fig. 3:**
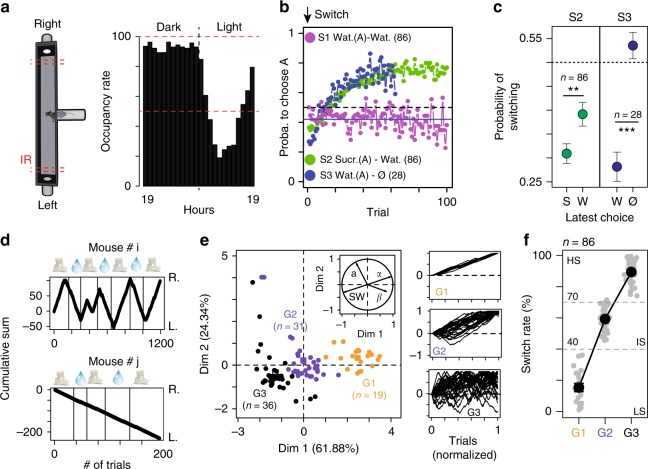
Decision-making. **a** T-maze occupancy (in %) on a 24 h cycle. **b** Probability to choose the highest rewarded arm (A) in sessions S1, S2, and S3. For S2 and S3, the first choice corresponds to the one after the bottles have been swapped. **c** Win-Stay strategy: probability to switch side when the latest choice (in *x*-axis) is sucrose (S) or water (W) for S2 and water (W) or nothing (N) for S3. (*W* = 4393 and 675.5, *p* = 0.0012 and *p* < 0.001). **d** Cumulative left (L.) or right (R.) turns for two different mice (# i and j), upon water and sucrose bottle swapping in S2 (symbols on top, indicating bottle content on the R. side). **e** Principal component analysis based on *a*, SW; *α* and *β*, (*n* = 86 from 9 experiments) from which we clustered three different groups (G1, G2, and G3). Insets on the right show normalized plots equivalent to **d**. **f** The three groups are well characterized by their difference in SW (i.e., low (LS), intermediate (IS), and high switch (HS) rates). Data **c**, **f** are presented as mean ± sem; ***p < 0.001, **p < 0.01, *p < 0.05

To refine individual description, we next addressed the relationship between social and non-social aspects of decision-making processes. In the T-maze with restricted access, mice voluntarily and individually performed a decision task, i.e., whether to make a left or right turn for accessing liquid reward. Once the choice for a particular arm (left or right) was made, the other arm closed off and the animal had to exit the test area for a new trial to resume (Supplementary Fig. 1D). The location of the different bottles was regularly swapped (every 3–4 days). The animal had thus to continually probe the environment and to adjust its behavior in response to changes in rewarding outcomes. The occupancy rate in the T-maze reflects circadian rhythms. It reached ~80% during the dark phase and dropped down to 20% during the light one (Fig. 3a, one experiment *n* = 18 animals). We then estimated, for the first 100 trials, the mean probability of choosing (i) the left arm in S1, (ii) sucrose in S2, and (iii) water in S3. For that purpose, we analyzed data from nine experiments with 86 mice. Three sessions were performed in each of these experiments (Ha, S1, and S2). For two of these experiments (*n* = 28 mice), a third session S3 was added. We found that mice preferentially chose the most rewarded side, i.e., sucrose for S2 and water for S3 (Fig. 3b). In S1, mice randomly opted for the two arms (i.e., 50% each) at the population level. The evolution of the probability to choose the best option after a bottle swap (Fig. 3b, green or blue curve) suggest a classical reinforcement-learning process for tracking the best-rewarded side by trial-and-error. In addition, at the population level, mice showed a decreased return time after choosing the less-rewarded side (Supplementary Fig. 5A) and used a win-stay strategy: they chose the same side after finding the best-rewarded side with high probability, but virtually chose randomly (i.e., around 50%) after missing it (Fig. 3c). A closer examination at the level of individual behaviors revealed that some mice did not alternate in S2 and thus failed to allocate their choices according to the location of the highest reward (Fig. 3d). To identify differences in behaviors, individual choice sequences were thus characterized by four variables that aimed to differentiate choice strategy. Two of these variables (*α* and *β*) were derived from modeling the choice sequence using a classical softmax model of reinforcement-learning/decision-making. The other two (switch rate noted SW, and slope a) were directly estimated from the choice sequence (see Methods). Clustering analysis (see Methods) distinguished three groups of mice (Fig. 3e): (i) G1 mice, characterized by a low switch and virtually no alternation, which always visited the same arm independently of the reward location; (ii) G2 mice, which are characterized by an intermediate behavior; and (iii) G3 mice, which consistently switched to track higher rewards. The low (LS), intermediate (IS), and high switch (HS) rates of the animals were found to be good indicators for distinguishing the three groups (Fig. 3f). Although the behavior of LS mice may appear suboptimal, this population emerged in most experiments (mean ± sem = 22.1% ± 7.5, *n* = 19/86 mice from nine experiments).

### DA neuron activity correlated with individual profiles

**Fig. 4:**
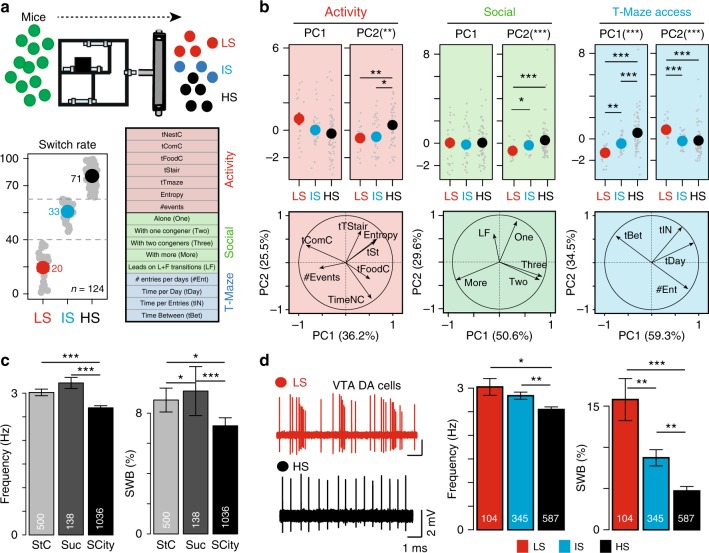
Correlation between specific cognitive behaviors and electrophysiological properties of the DA system. **a** Different groups of mice (10 groups, *n* = 124 mice) were tracked during the S2 in Souris City and classified according to their SW in three groups (i.e., low (LS), intermediate (IS), and high switch (HS) rates). Sixteen variables categorized in three groups were estimated: general activity, social, and T-maze access variables. **b** (Top) Correlation between principal component PC1 and PC2 for typical behaviors and SW. (Bottom) Correlation circle plots for the first two principal components (PC1 and PC2) with the different variables represented by their projections. (Left) General activity variables (*X*^2^ = 13.101, df = 2, *p*-value = 0.0014 for PC2). (Middle) Social variables (*X*^2^ = 14.972, df = 2, *p*-value = 0.00056 for PC2). (Right) T-access variables (*X*^2^ = 32.046, df = 2, *p*-value < 0.001 for PC1; *X*^2^ = 18.86, df = 2, *p*-value < 0.001 for PC2). **c** (Left) Spontaneous DA cell activity in standard cages with water (StC), sucrose (5%) or in Souris City (SCity) (frequency: *X*^2^ = 32.714, df = 2, *p*-value < 0.001; %SWB: *X*^2^ = 17.172, df = 2, *p*-value < 0.001). **d** (Left) Representative electrophysiological recordings of DA cells from LS (above) and HS mice (below). (Right) VTA DA neuron firing activity of the three groups (*X*^2^ = 13.601, df = 2, *p*-value = 0.0011 for frequency, *X*^2^ = 26.919, df = 2, *p*-value < 0.001 for %SWB). All Kruskal–Wallis were followed by a pairwise Wilcoxon post-hoc test with a Holm’s correction. All data are presented as mean ± sem, ***p < 0.001, **p < 0.01, *p < 0.05

After having revealed the existence of various profiles in Souris City, we next aimed at linking cognitive performances in the T-maze with traits derived from spontaneous behaviors and with individual neurophysiological activities. For that purpose, new experiments were performed, after which the electrophysiological status of each animal (10 groups, *n* = 124 mice) was analyzed. Sixteen variables were taken into consideration during S2. To avoid potential pitfalls associated with missed detections, variables derived from floor antennas (such as chasing episode) were not used here. The sixteen variables were then divided into three main classes: variables related to the general activity, social variables, and T-access variables (see Methods for the description of the different variables, Fig. 4a). In each class of variables, a principal component analysis (PCA) was performed and the first and second principal components were extracted. We found that the SW obtained in S2 (Fig. 4a) correlated with both social (Fig. 4b middle) and non-social variables (general activity (Fig. 4b, left) and T-maze access (Fig. 4b right)). This analysis suggest that LS mice spent more time in the nest and food sub-compartments (Fig. 4b, middle) and with groups of three or more congeners (Fig. 4b middle), but visited the test zone less frequently than the other groups (Fig. 4b, left). We then assessed whether these phenotypical differences correlated with physiological alterations of specific neural networks, and more specifically the mesolimbic DA system, which is often considered as an important player in personality neuroscience^21^. Variations in DA have indeed been observed across behavioral traits^22^. Moreover, this pathway was shown to encode the rewarding properties of goal-directed behaviors, including social interaction^23^, and to be a key system in stress-related disorders and addiction^24,25^. Importantly, repeated social defeats produces strong and long-lasting changes within the mesolimbic DA pathway, leading to social withdrawal of defeated individuals^14,26^. To address differences in the DA system between animals, we systematically recorded the activity of DA neurons following an experiment in Souris City. Mice were anesthetized and ventral tegmental area (VTA) DA cell activity was recorded using glass electrodes. DA cell firing was analyzed with respect to the average firing rate and the percentage of spikes within bursts (see Methods for burst quantification^27–29^). We first compared VTA DA cell activity in mice living in Souris City and in conspecifics living in a standard cage with access either to water (StC) or to a 5% sucrose drinking solution (Suc). Both the firing frequency and the bursting activity of VTA DA cells were significantly lower in Souris City compared to StC or Suc (Fig. 4c, left, Supplementary Fig. 6A–C). Furthermore, when analyzing separately the three groups of mice (LS, IS, HS), an inverted correlation between SW and both the frequency and bursting activity of VTA DA cells was observed (Fig. 4d, left and right, see also Supplementary Fig. 7A). Analysis of linear correlations between discharge rates (burst or frequency obtained per animal) and behavioral variables (Supplementary Fig. 7B–D) also indicated a link between VTA DA cell activity and behavior of the mice in the main environment. These results demonstrate a biological inscription, at the level of the midbrain DA system, of the stable and distinctive patterns of behavioral activity that emerged in this complex environment.

### Social relations shaped individual profiles and DA activity

**Fig. 5:**
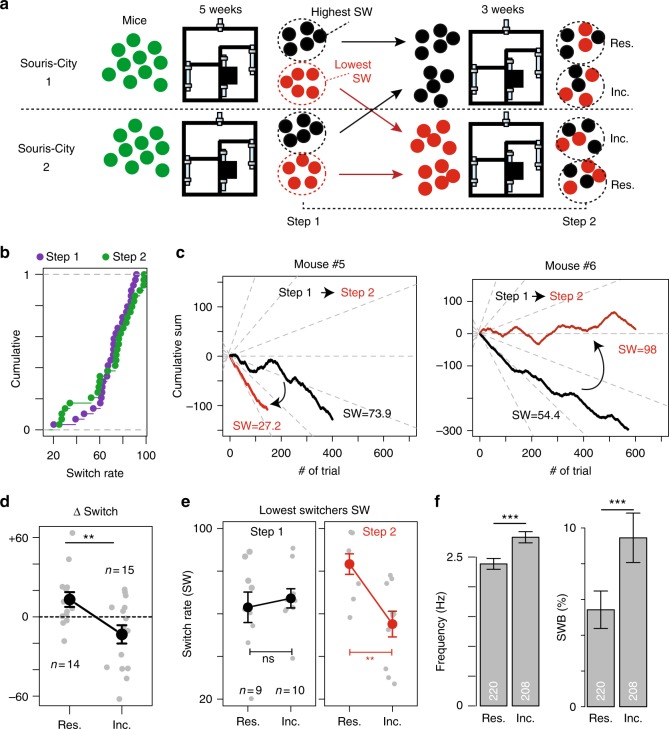
Influence of the group on individual behaviors and on the DA system. **a** Experimental paradigm: two different groups of mice were studied in parallel in two Souris City environments (1 and 2). After 5 weeks, their switching pattern were evaluated (step 1). Mice from each Souris City were split in lowest (red) and highest (black) switchers. The two populations were then mixed and the lowest switchers from the two environments were grouped together (same for the highest switchers). After three weeks of sucrose versus water, the switching patterns were re-evaluated (step 2) for both residents (Res.) and incomers (Inc.). **b** Cumulative distribution of SW for steps 1 (purple) and 2 (green) (*D* = 0.2069, *p* = 0.57). **c** Cumulative left or right turns for two different mice upon water and sucrose bottle swapping in step 1 (black) and step 2 (red). The incomer mouse #5 switched less, whereas the resident mouse #6 switched more in step 2 compared to step 1. **d** Switch variation between step 1 and 2 (∆SW) for incomers and residents (two-sample *t*-test, *t*(27) = 2.9401). **e** (Left) No difference in SW in step 1 between lowest switchers of the two different Souris City, whether they will be subsequently considered as incomers or residents. (Right) SW is different for the same two groups after step 2 (Two-sample *t*-test (*t* = 3.5914, **p < 0.01)). **f** Firing activity of VTA DA neurons from incomers and residents (frequency and SWB: two-sample Wilcoxon, *W* = 17,750 and *W* = 18,319 respectively, *p* < 0.001). ***p < 0.001, **p < 0.01, *p < 0.05

An important question remained, as whether these patterns were irreversible, i.e. related to intrinsic accumulated differences or, conversely, rapidly reversible. We addressed this issue by modifying the composition of two different groups of mice studied in parallel in two Souris City environments (Fig. 5a). During the sucrose versus water session, we used the median SW value to split mice from each Souris City in two populations: the lowest and highest switchers (step 1). We then mixed the two populations and grouped the lowest switchers from the two environments together, and the highest switchers together. After 3 weeks of sucrose versus water, we re-evaluated the switching pattern for each mouse (step 2). Interestingly, distinct switching profiles re-emerged within each of the two populations (HS, IS, and LS), with no significant difference in the overall distribution of SW before and after mixing (Fig. 5b). Individually, mice that had been relocated (referred to as incomers) to an unknown Souris City decreased their SW (e.g., mouse number #5 in Fig. 5c), whereas mice that did not move (referred to as residents) increased their SW (e.g., mouse number #6 in Fig. 5c). Variation of switching (i.e., SW_step2_ − SW_step1_) was higher in incomers than in residents (Fig. 5d). SW in step 1 was not predictive of SW in step 2: SW of the lowest switchers was homogenous in step 1 (Fig. 5e, left) but greatly diverged in step 2, with a clear SW difference between residents and incomers (Fig. 5e, right). Finally, we asked whether adaptation of SW was associated with a modification of VTA DA cell firing activity. DA neurons of incomers showed both higher firing rate and bursting activity than those of residents (Fig. 5f, Supplementary Fig. 8A). Altogether, these results suggest that the distinctive patterns of behavioral activity that emerged in this environment are rapidly reversible, and that social relationships can indeed shape individual behavior and affect the decision-making system.

## Discussion

Groups of mice have complex social structures^30^. Social interactions markedly influence a number of behaviors^17,31^, yet how they affect the development of inter-individual variability has rarely been addressed in standardized tests. Numerous studies emphasize the need to use large social housing environments, with automatic testing^20,32–35^. Such environments have up until now been mainly used to evaluate strain differences^18,19^ or test the effect of specific perturbations such as stress on subgroups^20^. An essential benefit of automation is that it challenges the classical paradigms consisting in the analysis of average behaviors in distinct groups of animals observed on a short timescale, and puts forward the statistical analysis of individuals recorded in an ecological situation over long timescales. In a relatively stable context, genetically identical animals adjust their behavior over time and situations, yet only within a given range, which is what defines individuality. In our experiments, the estimated variables may relate to either different traits or states of the animal, which refer to the notion of habitual versus transitory patterns of behavior, respectively^36^. Yet one key point here is that comparing mice allowed us to investigate individuality. We did not just measure the reaction of individuals to a new environment, we evaluated differences between individuals and stability of these differences when animals were faced with environmental changes. The notion of individuality thus challenges the idea that behavior of an individual is plastic and able to adapt optimally to its environment^1–4^. For instance, the fact that in our setting two individuals could be classified as either high or low switchers necessarily implies consistency in their decision-making system, and may reflect a limitation to their respective range of adaptation. Our results suggest that this limitation is, on the one hand, strongly linked to current social rules, as evidenced by the experiment during which we swapped social environments and, on the other hand, not influenced by local and immediate dynamics of social interactions, since the decision to switch is made in isolation from the congeners.

Initial variations on a small scale (developmental, epigenetic, or micro-environmental) have been proposed to support phenotypic variations on a large scale^12,37^. These small variations are believed to get amplified, resulting in a time-divergence of individual profiles, perhaps due to self-reinforcing effects of past experiences. In this framework, individuality emerges slowly and gradually, from small-scale initial individual variations to generate unique phenotypical trajectories. These assumptions do not necessarily imply that individual traits remains unchanged throughout life^38,39^. Our data shed new light on the role of social behaviors as a factor of divergence contributing to a reorganization of behavior. Social relationships are likely able to amplify initial differences between individual, but can also, as revealed here, trigger rapid and important reshaping of the individuality and of the DA system, through the dynamic effects of interactions between individuals. These results are compatible with the concept of social niches, which offers an adaptive explanation of the emergence of individuality based on specialization^3^. Yet, they also support the idea of a key social determinism, in which individuation is decisively determined by social processes and originates from the restriction of the animal capacities to a specific repertoire.

Variations in neuromodulatory functions, including those in the catecholamine and cholinergic systems, might contribute to the process of individuation^37,39^. DA produced in the VTA has a role in a wide range of behaviors, from processing rewards and aversion to attention, motivation, and motor control. The mesolimbic projections participate also in the modulation of social behaviors, as illustrated by genetics studies in human and physiological approaches in rodents^23^. In the course of a social interaction, an animal must be able to rapidly choose the appropriate behavior, for approaching or avoiding a conspecific. Previous studies demonstrated that the DAergic system undergoes activity-dependent changes^40^ that are triggered by events occurring during the lifespan of an individual^22,29^ and that affect basal activity in the long term. The modifications of DA cell activity observed in Souris City may reflect consequences of social events. Indeed, it has been shown that the regulation of the DAergic transmission is sensitive to social-stress exposure^26,41–44^. Alteration of DAergic activity has also been linked to many motor, motivational or cognitive dysfunctions. In particular, alteration of DA levels has been associated with variations in personal traits and, in the case of tonic DA, with exploration/exploitation trade-off or uncertainty seeking^45,46^. Furthermore, acutely manipulating VTA DA cell activity using optogenetics^47^ or pharmacology^26^, in the context of repeated severe social stress, is sufficient to reverse social-induced stress avoidance. All these results suggest a causal relationship between variations of VTA DA cell activity and the expression of specific behaviors.

Finally, our results open new perspectives for preclinical studies on rodent models. Preclinical models usually display high inter-individual variability, but do not focus on individuals. For instance, repeated social defeat in genetically identical mice leads to the appearance of depressive-like behavior only in a fraction of susceptible animals, but not in resilients^15,25^. Our results indicate that social relationships modify behaviors and circuits in a way that mimics the effects of certain mutations or drugs. The Souris City setup thus represents a unique opportunity to address causal relationships between cognitive performances in paradigms relevant for psychiatry and personality traits. Understanding how the social rules amplify the differences in behavioral spectrum displayed by otherwise identical animals will undoubtedly help unraveling the factors influencing the susceptibility of particular populations to psychiatric disorders.

## Methods

### Animals

Eight-week-old male C57BL/6J mice were obtained from Charles Rivers Laboratories, France. All procedures were performed in agreement with the recommendations for animal experiments issued by the European Commission directives 219/1990 and 220/1990 and approved by the Comité d’Ethique En Expérimentation Animale n°26. All mice were implanted under anesthesia (isoflurane 3%—Iso-Vet, Piramal, UK), with an RFID chip subcutaneously inserted in the back. For electrophysiological recordings following standard housing, mice are received at the age of 8 weeks, bred in cages of 5 for 2–4 weeks with water or sucrose solution (5% sucrose) before dopaminergic cell activity recordings.

### Souris City setup

Setup: Souris City combines a large environment (the social cage) where groups of male mice live for extended periods of time in semi-natural conditions, and a test zone where mice have a controlled access to specific areas for drinking. Souris City was house-designed and built by TSE Systems (Germany). Mice were tagged with RFID chips, allowing automatic detection and controlled access to the different areas. Animals were living under a 12 h/12 h dark-light cycle (lights on at 7am) and had access to food ad libitum.

The social cage is divided into four sub-compartments: NC, which contains a nest, FC where mice have free and uncontrolled access to food, CC and St to get access to the gate (Fig. 1a, Supplementary Fig. 1). NC, FC, and CC are located in a 1 m × 1 m square, on which St is connected by a tube. These different sub-compartments are equipped with RFID antennas on the floor and are connected through tubes that are also equipped with antennas. Therefore, each transition from one sub-compartment to another was associated with a detection of the animal by the two antennas of the transition tube. To avoid undetected transition due to high mouse speed, the diameter of each tube is reduced to 25 mm (Supplementary Fig. 2A). Our data did not reveal any undetected transitions (Supplementary Fig. 2B).

The social cage is connected to the test zone by a gate, which is a key element of the setup (Fig. 1A). The gate (TSE Systems, Germany) is composed of three doors with independent automatic control (Supplementary Fig. 1B), allowing to select animals and control their access to the test zone. Individuals thus performed the test alone (isolated from their congeners) and by themselves, i.e., whenever they wished to and without any intervention from the experimenter. The test consists in a T-maze choice task^48^. Since the T-maze was the only source of water, animals were motivated to perform the test. The T-maze gives access to two home-cages, one on each side (left and right), with a drinking bottle in each. The bottles contained either water, sucrose or were empty. The system was configured in such a way that animals performed a dynamic foraging task. The reward value of the bottle content could be changed, to evaluate whether mice were able to track the highest reward. Such automation of the task, by minimizing handling and the presence of the experimenter, prevents most limitations of human assessment (i.e., cost and time) and eliminates the risks of stress or disturbance of the animal natural cycle^20,33,34,49^. Simple rules were used to automatize the test. When a mouse accessed one feeder, the infrared light beam was cut off in that arm, which triggered closing of the feeder on the other side (a Plexiglas cylinder drops in and prevents access to the bottle). Mice had to exit the T-maze to trigger re-opening of the feeders and hence to resume a new trial (Supplementary Fig. 1D). Bottles (for example sucrose- or water-containing) were swapped every 3–4 days.

Event detection and storage: Four different kinds of sensors provided automatic data registration in Souris City: RFID antennas surrounding the tubes that connect sub-compartments together (*n* = 14), the gate (*n* = 1), infrared beam sensors in the T-maze (*n* = 4, 2 on each side), and RFID antennas on the floor (*n* = 16). The IntelliMaze software (TSE Systems, Germany) ran the first three sensors, while TraffiCage (TSE Systems, Germany) controlled the floor RFID antennas. These two software programs worked completely independently. IntelliMaze registered a table (.txt file) for each sensor, where each line corresponds to a detection event with the information on animal identity (RFID tag), detection time (millisecond precision), antenna number for the tubes and animal direction for the gate. The detection range was the distance between the center of the antenna and the point where the RFID chip was first detected when approached gradually to the center of the antenna (precision: one millimeter; Supplementary Fig. 2A). Detection reliability was estimated to be 100% for the tube-antennas (Supplementary Fig. 2B), indicating a very high confidence for the estimation of the presence of an individual within a given sub-compartment. With respect to the reliability of detection at the level of the floor antennas, our data indicate that animals were detected 75% of the time when their trajectories crossed the antenna. We did not record any false detections (Supplementary Fig. 2B). Non-detection could be due to (i) the animal bypassing the detection area of a given antenna, (ii) the animal not being detected due to its speed, (iii) multiple animals being present on the same antenna (in case where two mice are simultaneously present on a given antenna, only one is detected). The TraffiCage software registered detection events as a raw file (.txt file) with the information of animal identity, detection time, and antenna number. All these detection events were stored in a database (MySQL relational database hosted by an Apache server), together with spatial and temporal annotation allowing to track the position and activity for each mouse (i.e., mouse number, date, time, antenna number). A web interface coded in php imported the data from the files into the database, linked all the events to the appropriate mouse and created gate sessions. All these events constitute the basic data used for further analysis (see data analysis). R scripts (RMySQL package) were used to extract data from the database.

Data processing: Detection events were used to build various indices and estimators of the animal behavior. The position of the animal was used to calculate its overall activity: (i) the proportion of time spent in each sub-compartment, (ii) the density of transitions between sub-compartments computed on 24 h, binned by 10 min periods to evaluate the circadian rhythm, (iii) the number of detections for each antenna, and (iv) the entropy of each animal. Entropy was calculated from the proportion of time *p* spent in each sub-compartment *i*:

1 $${\mathrm{Entropy}} = - \mathop {\sum }\limits_i p_i\log (p_i)$$

The localization of a mouse relative to others was used to assess the social relationships between mice, e.g., the proportion of time spent alone, with one conspecific or more. We also quantified for each mouse the number of times that a transition, from one compartment to another, precedes or follows the transition of another mouse within a five seconds window (Fig. 1d). From these data, we estimated a lead ratio defined by LF = number leads/(number of leads + follows). We also used detections from both tubes and floor antennas to quantify chasing episodes between two mice. Chasing episodes were defined by concomitant (i.e., within a 5 s window) detections of the same two mice on at least two consecutive antennas. Antennas were considered consecutive if the first mouse from a concomitant detection on one antenna was detected within a 30 s window on another antenna (see Fig. 1e for schematics). Because the floor antenna system is not fully accurate and may fail to capture mice crossover (Supplementary Fig. 2), measures derived from floor detections were only used to highlight animal consistency (Fig. 2) but were not used in subsequent analyses (i.e., Figs. 3, 4, 5). Cumulative curves (entropy and time spent in FC) over sessions represent data from dark phase section (from 7pm to 7am the following day) summed with data from the dark section of the previous days. We categorized the set of variables (16 variables) into three domains: (Fig. 4a): the general activity (proportion of time spent in each sub-compartment, entropy, number of tubes detections per day), the social variables (time spent alone, with one, two, three or more other mice, LF), and T-access variables (number of entries, time per day, time per entry, time between two entries).

The T-maze choice quantification: Individual choice sequences (i.e., left or right, Fig. 3) were characterized using four parameters: the switch rate (SW, see above), the slope of the left-right choice (a value close to 1 indicating no switching), the exploratory parameter (*β*) and the learning rate parameter (*α*). We calculated SW for each animal as follows:

2 $${\mathrm{Switch}}\,{\mathrm{rate}} = 100 - \left| {\left( {\left( {\frac{{{\mathrm{Number}}\,{\mathrm{of}}\,{\mathrm{left}}\,{\mathrm{side}}}}{{{\mathrm{Total}}\,{\mathrm{number}}\,{\mathrm{of}}\,{\mathrm{trial}}}} \times 100} \right) - 50} \right) \times 2} \right|$$

A SW of 100% indicates that the mouse equally chose both sides, while a SW of 0% means that the mouse never switched and always chose the same side. Exploration/exploitation parameters were calculated by fitting the sequence of choices with a standard reinforcement-learning/decision-making model. We used a classical softmax decision-making model where choices depend on the difference between the expected rewards of the two alternatives. This model formalizes the fact that the larger the difference in rewards is, the higher the probability to select the best option will be. Sensitivity to reward difference was formalized by the free parameter *β*. Expectation of reward was adapted through classical reinforcement-learning algorithm, i.e., trial-and-error, by comparison between the current estimate of action; with *R*(water) = 1, *R*(sucrose) = 2, *R*(nothing) = 0. The value *V*_*i*_ of each action *i* was updated by *V*_*i*_(*t* + 1) = *V*_*i*_(*t*) + *αR*(*t*), where the free parameter *α* formalizes the learning speed. The softmax choice rule was:

3 $$P_i = \frac{{{\mathrm{exp}}(\beta V_i)}}{{\mathop {\sum }\nolimits_j {\mathrm{exp}}(\beta V_j)}}$$

where *β* is an inverse temperature parameter reflecting the choice sensitivity to the difference between decision variables: high *β* corresponds to mice that often choose what they estimate the highest-value arm, while low *β* corresponds to random choice. The free parameters *α* and *β* were optimized using the log-likelihood of the model, on a choice-by-choice basis.

### Behavioral experiments

The system consists in two parallel and identical setups (Fig. 1a, Supplementary Fig. 1) enabling the analysis of groups of mice (see tab below). In this study, 14 experiments were performed, 12 of which were paired, i.e., executed in parallel in two independent setups. Two setups were physically coupled (at the St level) for a single experiment, which allowed the tracking of 18 mice. This experiment was used to illustrate some typical results on a larger group of mice (Fig. 2a–c). Overall, 135 mice were tested in Souris City. In one experiment, one mouse was not drinking and was rapidly excluded. The first array indicates the experiment numbers and the number of mice used in the analysis Table 1.

**Table 1:** Number of mice per experiment

Experiment #	1	2	3	4	5	6	7	8	9	10	11	12	13	14
Number of mice	10	10	10	9	10	18	5	6	8	10	10	9	10	10

Experiments were not all pooled together because not all measures were made at all time (see Table 2).

**Table 2:** Experiments used in each figure

Figure #	Experiment #	Number of mice
Fig. 1	1, 2, 3, 4, 5	49
Fig. 2a–d	6	18
Fig. 2e–g	1, 2, 3	30
Fig. 3	1, 2, 3, 4, 5, 6, 7, 8, 9	86
Fig. 4	1, 2, 3, 4, 5, 6, 9, 10, 11, 12, 13, 14	124
Fig. 5	12, 13, 14	29

### In vivo electrophysiological recordings

Mice were anesthetized with an intraperitoneal injection of chloral hydrate (8%), 400 mg/kg, supplemented as required to maintain optimal anesthesia throughout the experiment, and positioned in a stereotaxic frame (David Kopf). Body temperature was kept at 37 °C by means of a thermostatically controlled heating blanket. All animals had a catheter inserted into their saphenous vein for i.v. administrations of drugs. Recordings were performed using classical technics commonly used in the laboratory^28,50^. Briefly, recording electrodes were pulled with a Narishige electrode puller from borosilicate glass capillaries (Harvard Apparatus). The tips were broken under a microscope. These electrodes had tip diameters of 1–2 mm and impedances of 20–50 MΩ. A reference electrode was placed into the subcutaneous tissue. When a single unit was well isolated, the unit activity digitized at 12.5 kHz was stored in the Spike2 program (Cambridge Electronic Design, UK). The electrophysiological characteristics of VTA DA neurons were analyzed in the active cells encountered by systematically passing the microelectrode in a stereotaxically defined block of brain tissue including the VTA. Its margins ranged from 3 to 3.8 mm posterior to Bregma, 0.25 to 0.8 mm mediolateral with respect to Bregma, and 4.0 to 4.8 mm ventral to the cortical surface according to the coordinates of Paxinos and Franklin^51^. Sampling was initiated on the right side, and then on the left side. After a baseline recording of 10–15 min, the electrode was moved to find another cell. Extracellular identification of DA neurons was based on their location as well as on a set of unique electrophysiological properties that characterize these cells in vivo: (i) a typical triphasic action potential with a marked negative deflection; (ii) a characteristic long duration (>2.0 Ms); (iii) an action potential width from start to negative through >1.1 Ms; (iv) a slow firing rate (<10 Hz and >1 Hz) with an irregular single spiking pattern and occasional short, slow bursting activity. These electrophysiological properties distinguish DA from non-DA neurons^27^. In our experiments, we also perform juxtacellular labeling of 114 neurons with neurobiotin. All of them, including few cells with a firing rate below 1 Hz, were identified as DAergic using Tyrosine hydroxylase (TH) immunochemistry.

### DA cell firing analysis

DA cell firing was analyzed with respect to the average firing rate and the percentage of spikes within bursts (%SWB, number of spikes within burst divided by total number of spikes). Bursts were identified as discrete events consisting of a sequence of spikes such that its onset is defined by two consecutive spikes within an interval <80 ms and its termination by an inter-spike interval >160 ms^27–29^. For each recorded neuron, the mean firing frequency and mean bursting activity were evaluated on a basis of a least 10 min of recordings. These mean values were used to characterize each neuron. Animal firing activity was estimated by pooling the activity from each neuron recorded in a given animal and estimated by a mean value.

### Statistics

Data are presented as means ± SEM with corresponding dot plots overlaid, as cumulative distribution function, or as boxplot. Data from electrophysiological recording (Fig. 4) are presented as barplot (mean ± sem) without dot plots, and their cumulative distributions are presented in supplementary figures (Supplementary Figs. 5, 6). Statistics for behavioral experiments were carried out using R, a language and environment for statistical computing (2005, www.r-project.org). We used a one-way repeated-measures ANOVA followed by a *t*-test with Bonferroni correction for post-hoc analysis to compare the time spent in each sub-compartment through several sessions (Fig. 2c). Consistency over two sessions was estimated by Spearman correlation coefficient (rho) between several measurements (e.g., proportion of time spent in the sub-compartments) determined in session S1 and S2 (Fig. 2d–g). Probability of switching were evaluated using repeated trials (i.e., consecutive entries with a maximum of 20 s apart) and were compared using two-sample Wilcoxon test (Fig. 3c). We performed a clustering (bclust function from e1071 package: https://cran.r-project.org/web/packages/e1071) and a Principal Component Analysis (PCA function from FactoMine package: https://cran.r-project.org/web/packages/FactoMineR) to define three groups of mice from the T-maze scores (Fig. 3e). PCA function from FactoMine package was also used to analyze behavioral variables (Fig. 4a, b). To compare group of samples (LS, IS, HS groups) we used a one-way ANOVA followed by a Tukey test (TukeyHSD test in R) for post-hoc analysis if data were normally distributed. If data were not normally distributed or variance not equal, we performed the Kruskal–Wallis test for multiple samples. This test was followed by a post-hoc test, in this case Wilcoxon rank test with Holm’s sequential Bonferroni *p*-value correction (Fig. 4). We calculated the difference between the SW before and after mixing the mice and we compared the incomers with the residents with a *t*-test or a Wilcoxon test depending on the distribution normality (Fig. 5d, e). The firing rate and %SWB of DA neuron were compared between these two groups with a Wilcoxon test (Fig. 5f).

### Data availability

The data that support the findings of this study are available from the corresponding author upon reasonable request.

## Electronic supplementary material

Supplementary Information

Peer Review Fie
