# Supplementary material for: Social interactions impact on the dopaminergic system and drive individuality
Source: Nat Commun. 2018 Aug 6;9:3081. doi: 10.1038/s41467-018-05526-5 (PMC6079008; doi:10.1038/s41467-018-05526-5)
Supplement: Supplementary file 1 — Supplementary Information [file 41467_2018_5526_MOESM1_ESM.pdf]

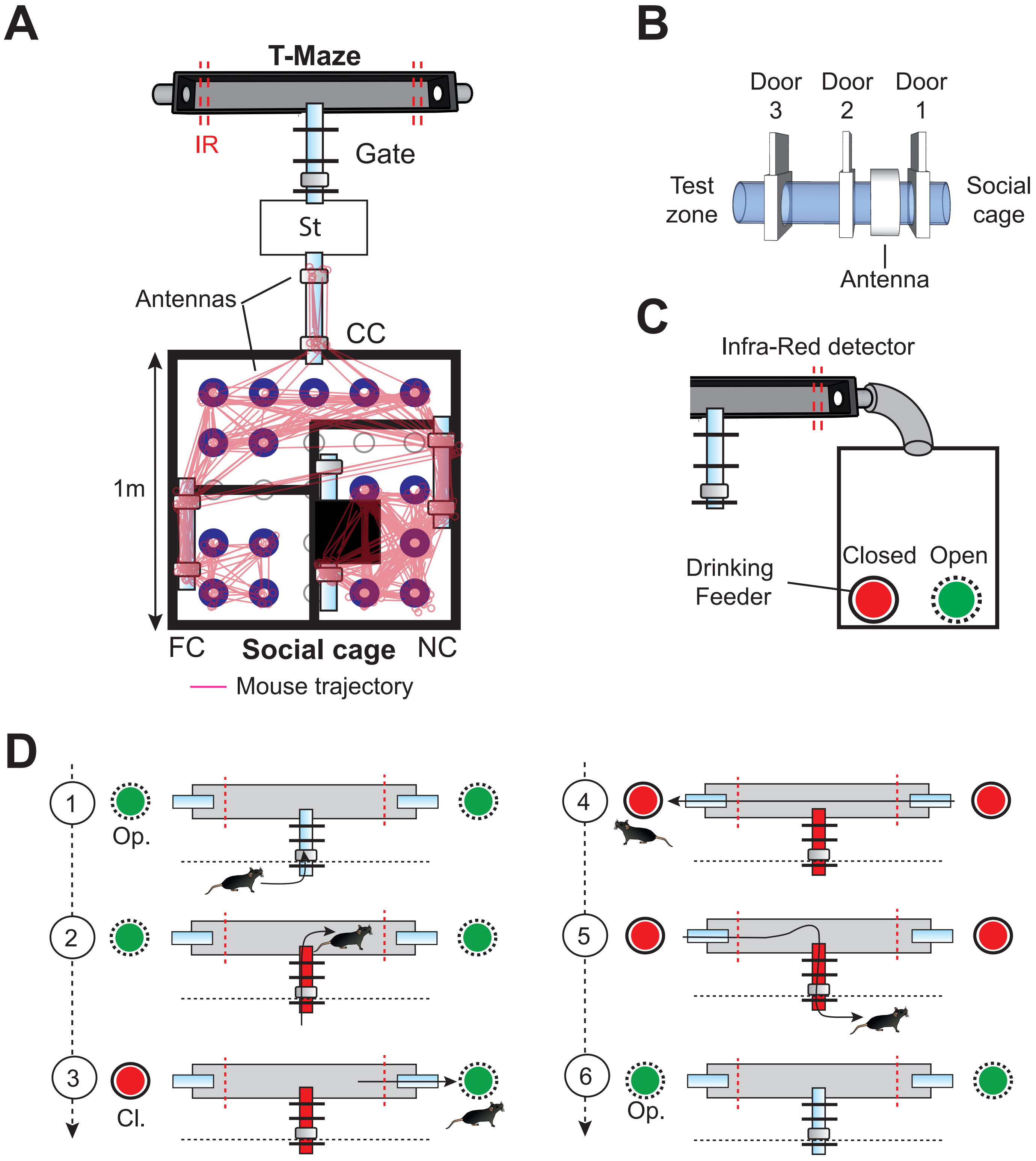

A
T-Maze
IR
Gate
St
Antennas
CC
1m
FC
Social cage
NC
Mouse trajectory
B
Door 3
Door 2
Door 1
Test zone
Social cage
Antenna
C
Infra-Red detector
Drinking Feeder
Closed
Open
D
1
Op.
2
3
Cl.
4
5
6
Op.

Supp Fig 1

Supplementary Figure 1: Souris City environment: (A) The Souris City system superimposed with an example of a trajectory based on 200 floor and tube RFID detections (pink). **(B) The gate** (TSE system) is composed of three doors with independent control. **(C) The drinking area:** The maze gives access to two home-cages (left and right) with one bottle in each. **(D) The test zone:** When a mouse enters the T-maze (Fig. 1D step 1), the gate closes and prevents T-maze access for other mice (step 2). Once the mouse makes a turn and cuts off the infrared detector line, access to the bottle on the other side closes off (step 3). The animal has then free access to the bottle content as long as it does no cut off the IR beam in the opposite arm of the T-maze (step 4), in which case access to both bottles gets prevented. The animal has to exit the test area (step 5) to resume a trial. The test area then becomes accessible to any other mouse (step 6).

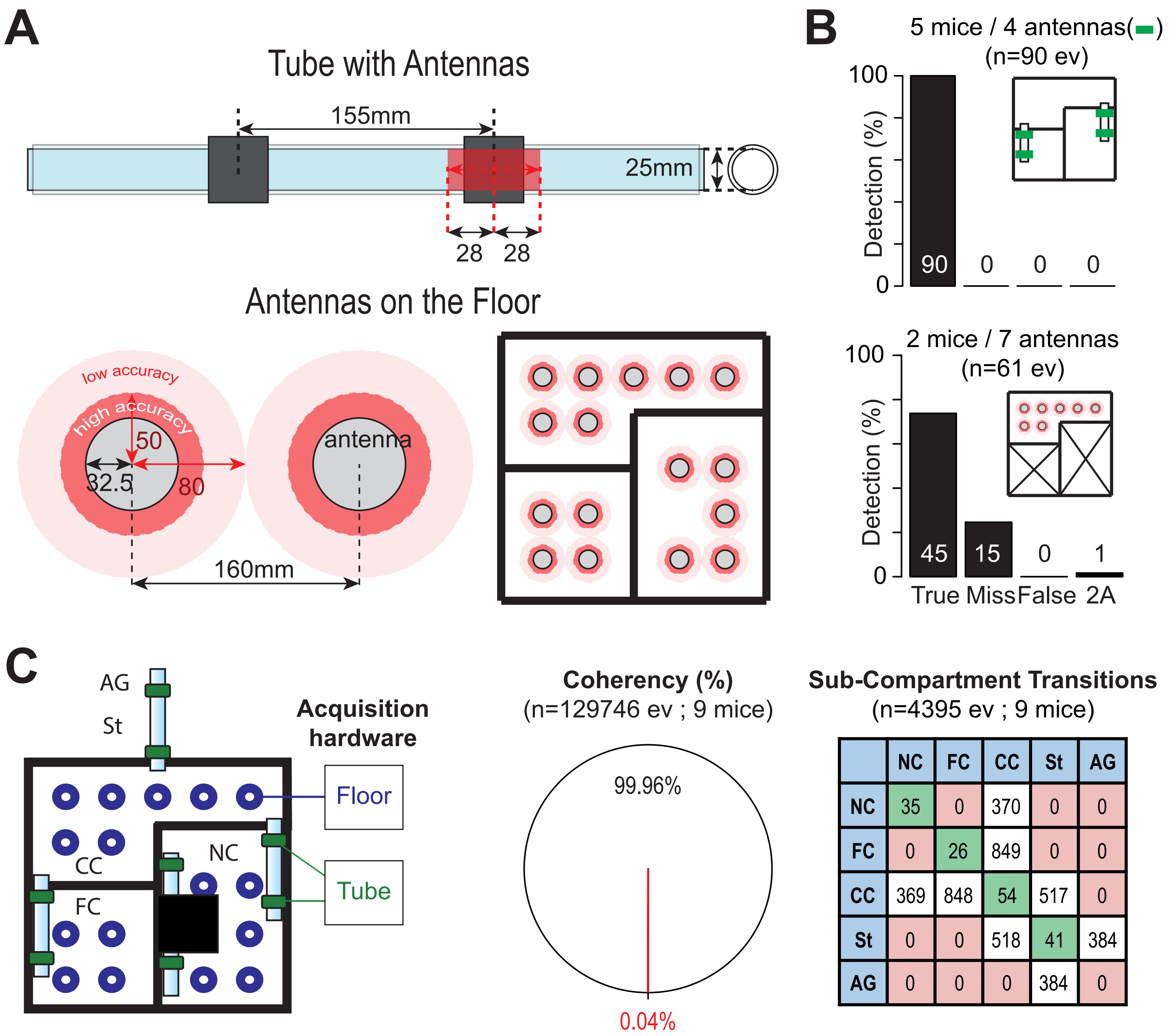

	NC	FC	CC	St	AG
NC	35	0	370	0	0
FC	0	26	849	0	0
CC	369	848	54	517	0
St	0	0	518	41	384
AG	0	0	0	384	0

Supp Fig 2

Supplementary Figure 2: Reliability of RFID detection. A) Antennas used to identify and locate each mouse. Detection is based on two independent systems: (Top) Tube antennas have a detection range of 28 mm from the center. (Bottom) Floor antennas have an estimated distance for accurate detection of about 50 mm from the center of the antenna, but some events can be detected at up to 80 mm from the center. A total of 16 antennas were distributed within the different sub-compartments. **B) Estimation of the reliability of detection.** Animals were introduced in a reduced field (inset) and the session was video-recorded in order to check RFID detections. (Top) 5 animals were introduced in a field with access to three sub-compartments through two tubes with four antennas (two per tube) during two hours. All of the transitions (45 transitions, i.e. 90 detections) from one sub-compartment to another were detected by the antennas. (Bottom) Two mice were placed in a sub-compartment (7 antennas) during ten minutes and were detected 75% (n=61 events) of the time when their trajectories crossed the antennas. No false detection was observed and one simultaneous detection on two distinct antennas was obtained. **C) Cross-validation of detection during a complete session (n=9 mice, 7 days).** (Left) The two RFID systems (tubes and floor antennas) are completely independent: each system is controlled by its own hardware and acquisition software (see methods). (Middle) Comparison of tracking and detection in a given sub-compartment by the two systems. Circular plot representing the percentage of incoherent detection at the level of the floor antennas, n=12746 detections on the floor, 99.96 % of these detections fit with the current location of the mouse in a given sub-compartment. (Right) Matrices of transitions between sub-compartments show the total absence of forbidden sequences (e.g. a direct transition between two unconnected sub-compartments, red in the matrices). In white, number of transitions between two connected sub-compartments. In green, cases where the mouse made a U-turn within the tube, prior to being detected by the second antenna of the tube.

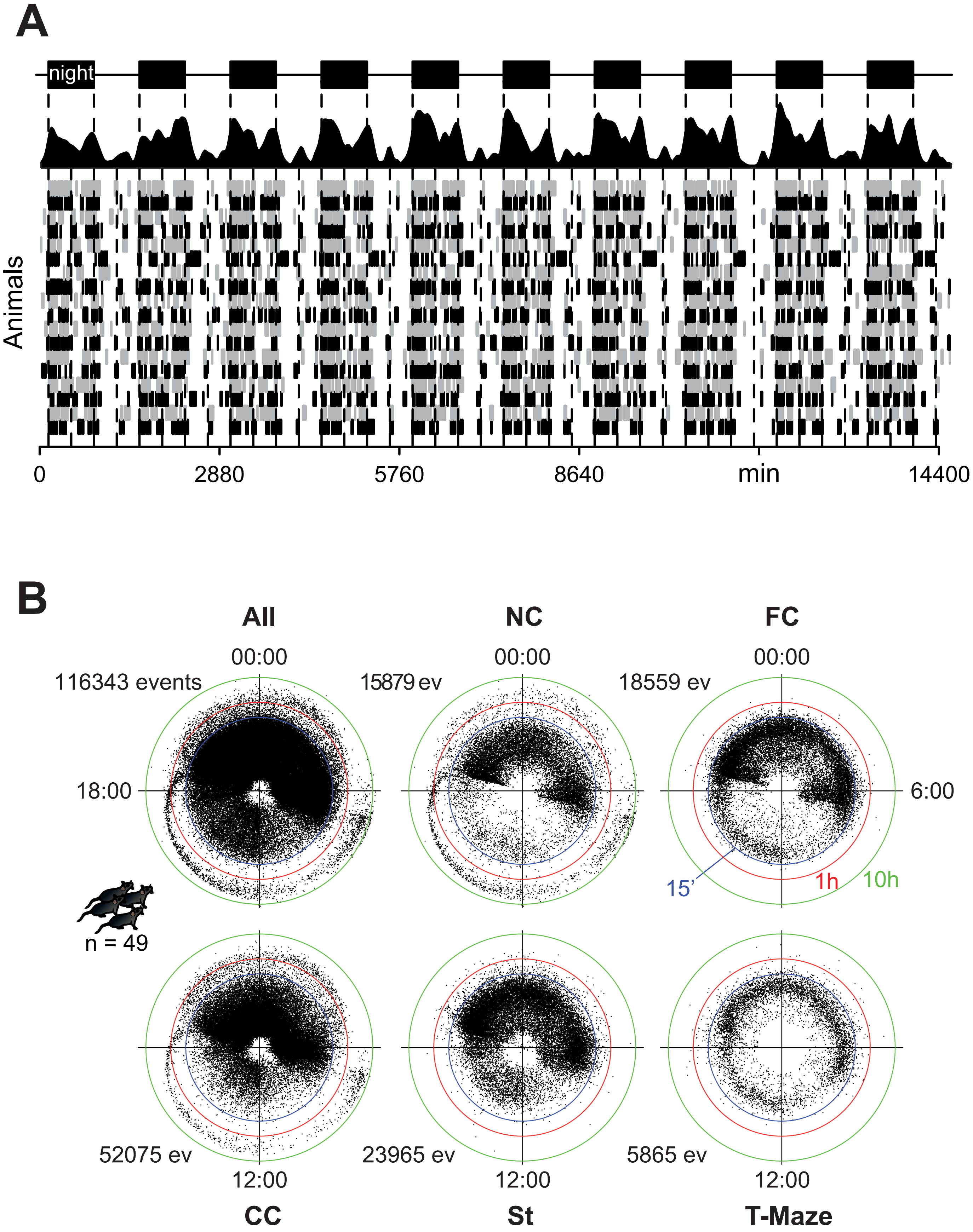
A
night
Animals
0
2880
5760
8640
min
14400
B
All
NC
FC
00:00
116343 events
15879 ev
18559 ev
18:00
6:00
15'
1h
10h
n = 49
52075 ev
23965 ev
5865 ev
12:00
CC
St
T-Maze

Supp Fig 3

Supplementary Figure 3: Tracking events in Souris City: (A) 10 consecutive days of activity for n=18 mice recorded simultaneously. Each vertical bar indicates one detection event for a given mouse (for this plot, only the tube detections are being considered). Density of activity for all the mice revealed a marked circadian rhythm. Day-night alternations are indicated on top. **(B)** Circular plots showing the detection time (each point) and duration of each visit (log distance of the point from the center) during the day in the entire Souris City (above left) and in the different sub-compartments. Note that very long stays, over 1 hour (red circle), were found for NC and CC. In contrast, very few events were longer than 15' in FC and in the T-maze.

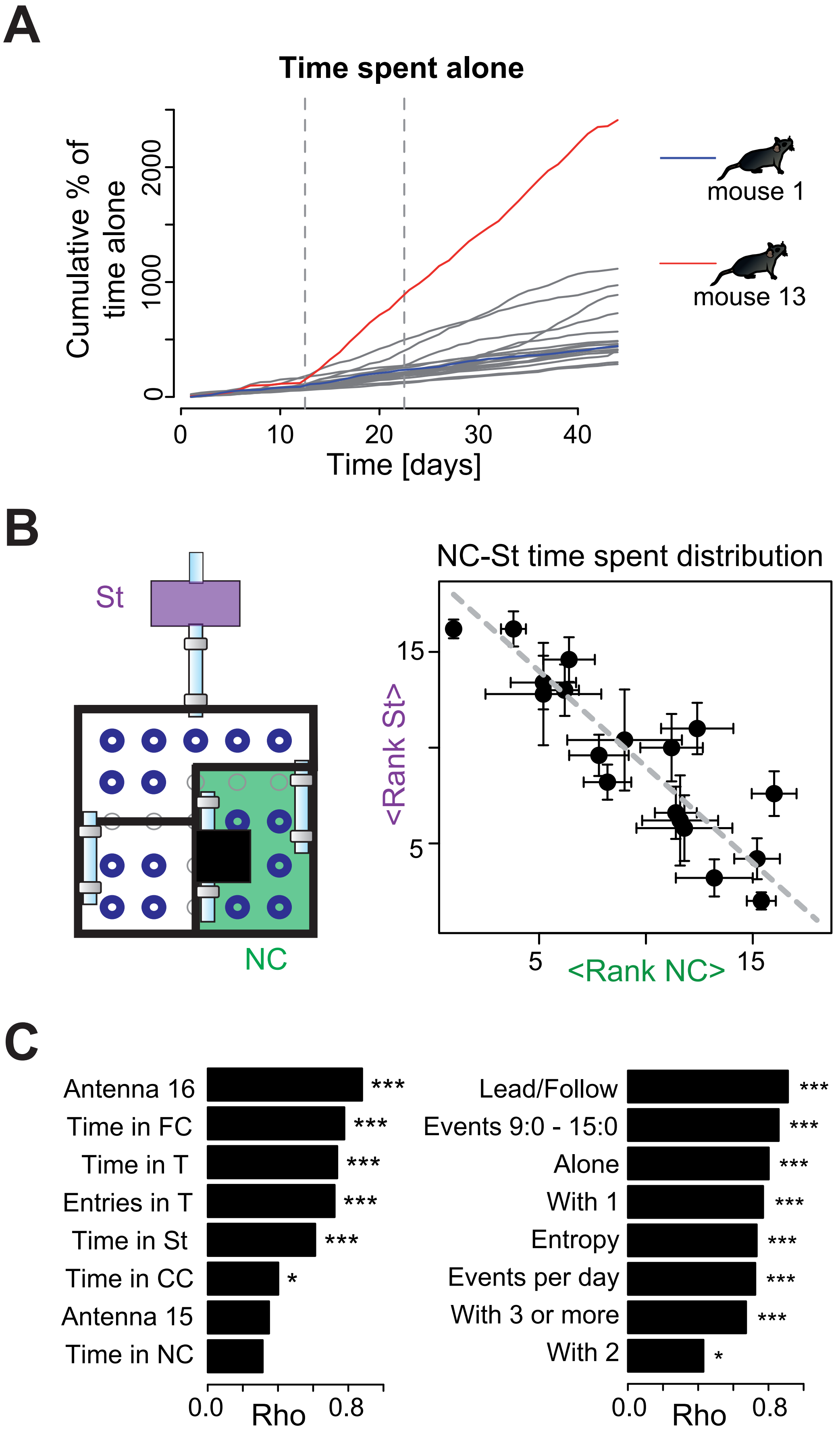
A
Time spent alone
Cumulative % of time alone
0
1000
2000
0
10
20
30
40
Time [days]
mouse 1
mouse 13
B
St
NC
NC-St time spent distribution
15
5
<Rank St>
5
<Rank NC>
15
C
Antenna 16 ***
Time in FC ***
Time in T ***
Entries in T ***
Time in St ***
Time in CC *
Antenna 15
Time in NC
0.0
Rho
0.8
Lead/Follow ***
Events 9:0 - 15:0 ***
Alone ***
With 1 ***
Entropy ***
Events per day ***
With 3 or more ***
With 2 *
0.0
Rho
0.8

Supp Fig 4

Supplementary Figure 4: Individuation: (A) Divergence of the cumulative of individual measures derived from the time spent alone. Note the divergence for mice #1 and #13. **(B)** Stability of behavior: Time spent in NC (green area) and St (purple area) were highly correlated. (Right) Mean rank observed in session 1 to 4 for n=18 mice. (mean ± sem). **(C)** Estimation of rho, the Spearman's rank correlation coefficient calculated on the rank on two consecutive periods for several behaviors, $p<0.001$ with a spearman test. Antenna 16: number of detections on antenna #16 (floor antenna in the top right of the social cage, in CC); Time in FC: time spent in FC; Time in T: time spent in the T-Maze; Entries in T: number of entries in the T-Maze; Time in St; Time in CC; Antenna 15: number of detections on antenna #15 (located on the left of antenna #16, in CC); Time in NC; Lead / Follow (see Fig. 1E); Events 9:0-15:0: number of events between 9:00 and 15:00; Alone: time spent in a sub-compartment with no other mice; With 1: time spent in a sub-compartment with one other mouse; Entropy (see methods); Events per day: number of events per day; With 3 or more: time spent in a sub-compartment with 3 or more mice; With Two: time spent in a sub-compartment with two other mice. See also Fig. 2G for some of these measurements. All these data are normalized with the duration of the session.

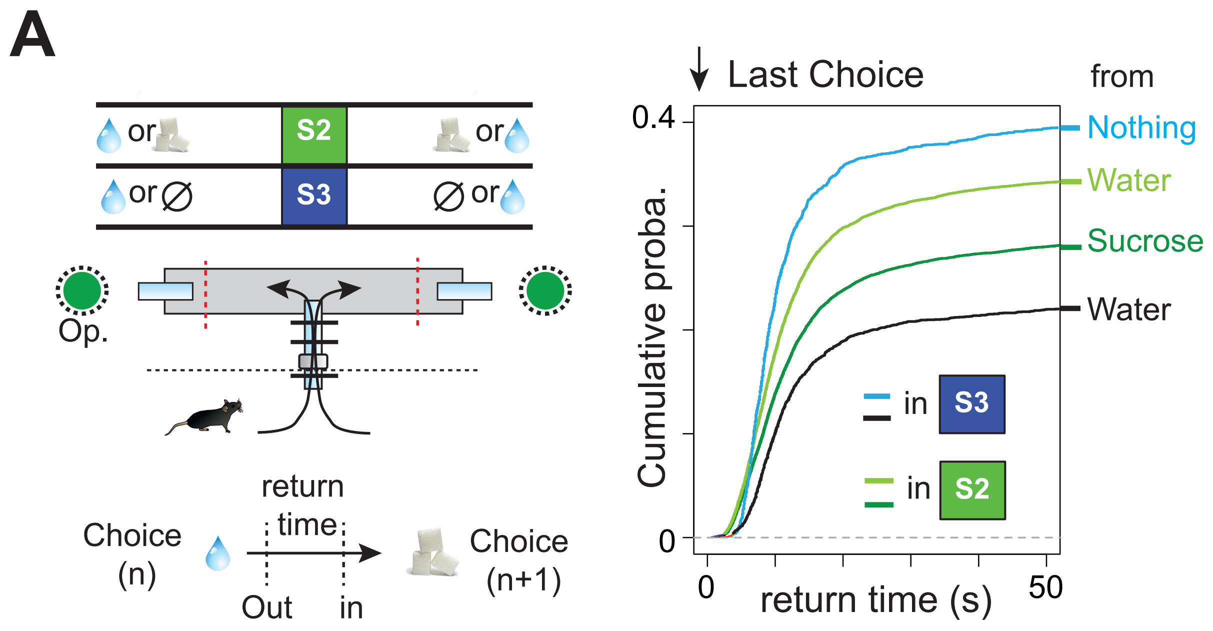
A
or
S2
or
or
S3
or
Op.
return
time
Choice
(n)
Out
in
Choice
(n+1)
Last Choice
from
0.4
0
Cumulative proba.
return time (s)
50
Nothing
Water
Sucrose
Water
in
S3
in
S2

Supp Fig 5

Supplementary Figure 5: Decision-making: (A) (Top left) Comparison of the return time in session S2 (water versus sucrose) and S3 (water versus nothing). (Bottom left) Return time was defined as the time between two consecutive entrances in the T-maze for a given mouse (time between the end of a trial (n) and the beginning of the next trial (n+1)). (Right) Cumulative density of the return time depending on the session and the last choice of the animal (i.e. water in S3 (Black line); Nothing in S3 (blue); sucrose in S2 (dark green); Water in S2 (light green)).

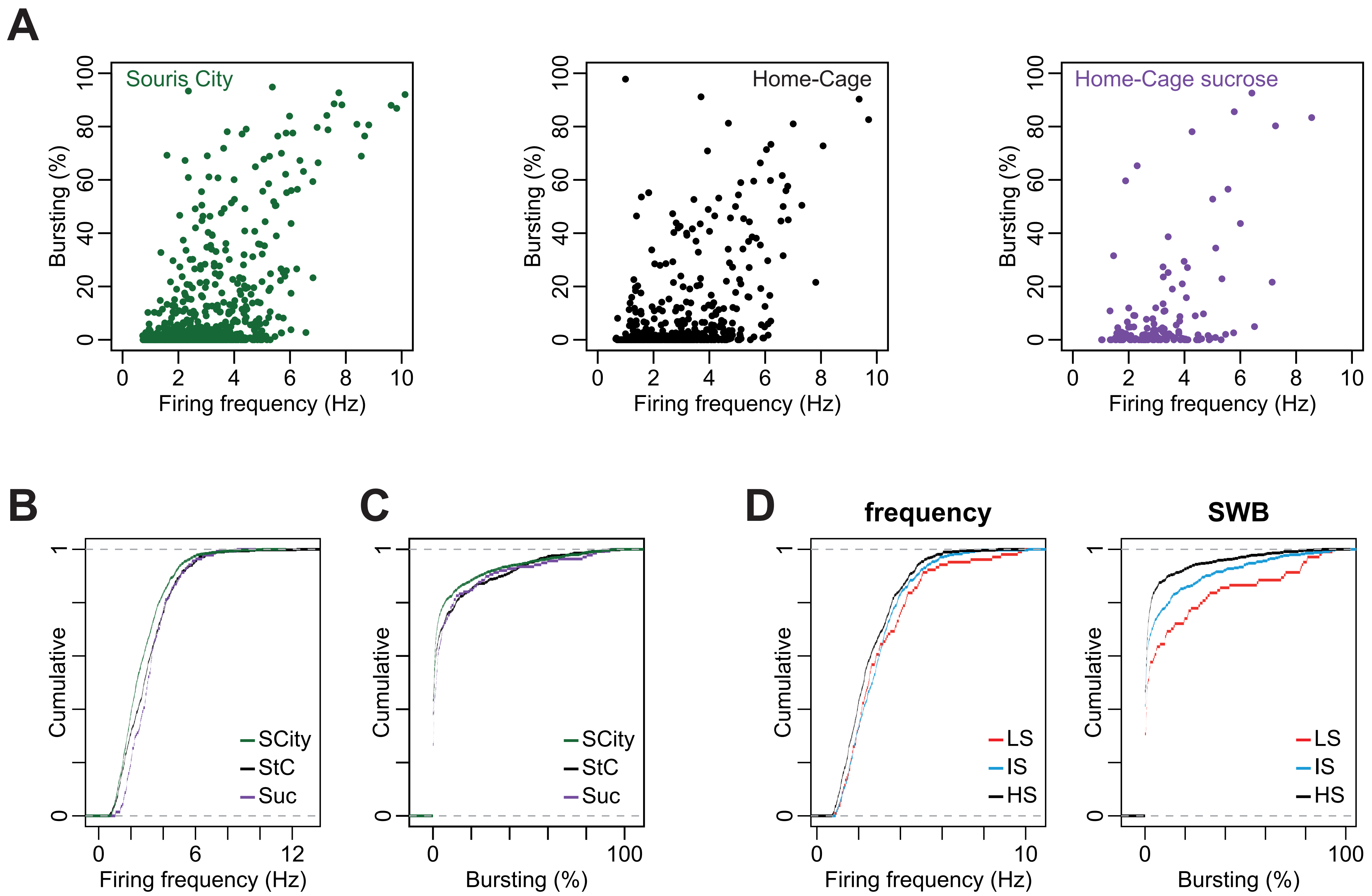
A
Souris City
Home-Cage
Home-Cage sucrose
Bursting (%)
Firing frequency (Hz)
B
C
D
frequency
SWB
Cumulative
SCity
StC
Suc
LS
IS
HS
Bursting (%)
Firing frequency (Hz)

Supp Fig 6

Supplementary Figure 6: ***In vivo*** **electrophysiology: (A)** (left) Plot of mean firing frequency (Hz) against percentage of spikes within a burst (%SWB) for WT mice either bred in Souris City (n=1280 cells, green), in classical laboratory cages (n=500 cells, black) or in classical laboratory cages with sucrose 5% instead of water (n=138 cells, purple). **(B)** Cumulative distribution of cell firing frequency and **(C)** of cell bursting (green: Souris City, black: classical cage, purple: classical cage with sucrose). **(D)** Cumulative distribution of cell firing frequency and of cell bursting for each group of mice (LS, IS, HS).

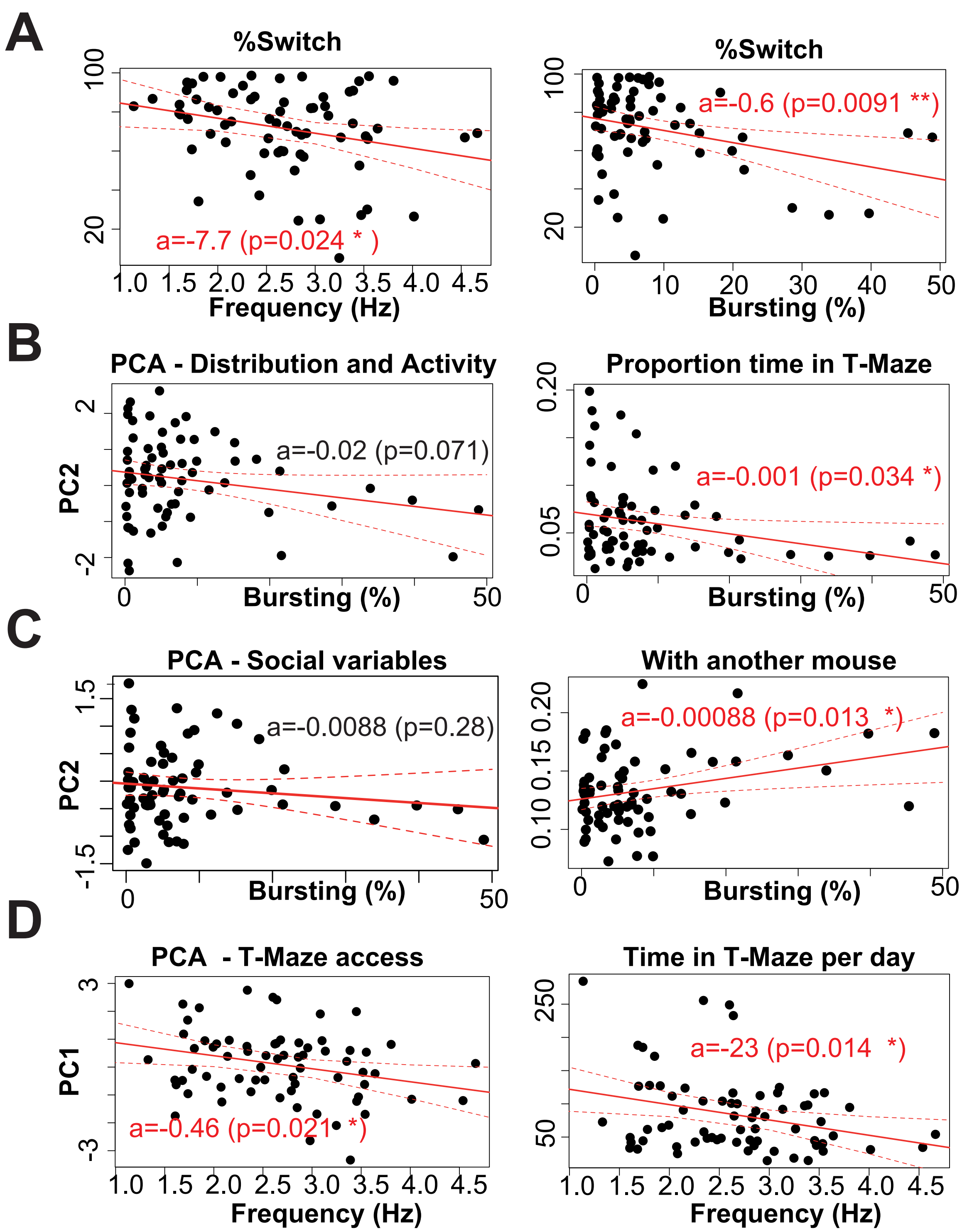

A
%Switch
100
20
a=-7.7 (p=0.024 *)
1.0 1.5 2.0 2.5 3.0 3.5 4.0 4.5
Frequency (Hz)
%Switch
100
20
a=-0.6 (p=0.0091 **)
0 10 20 30 40 50
Bursting (%)
B
PCA - Distribution and Activity
PC2
2
-2
a=-0.02 (p=0.071)
0 Bursting (%) 50
Proportion time in T-Maze
0.20
0.05
a=-0.001 (p=0.034 *)
0 Bursting (%) 50
C
PCA - Social variables
PC2
1.5
-1.5
a=-0.0088 (p=0.28)
0 Bursting (%) 50
With another mouse
0.10 0.15 0.20
a=-0.00088 (p=0.013 *)
0 Bursting (%) 50
D
PCA - T-Maze access
PC1
3
-3
a=-0.46 (p=0.021 *)
1.0 1.5 2.0 2.5 3.0 3.5 4.0 4.5
Frequency (Hz)
Time in T-Maze per day
250
50
a=-23 (p=0.014 *)
1.0 1.5 2.0 2.5 3.0 3.5 4.0 4.5
Frequency (Hz)

Supp Fig 7

Supplementary Figure 7: (A) Linear regression model between average in vivo VTA DA neuron firing frequency (left) or bursting (right) and %Switch from individual mice (R^2=0.06, p-value=0.02415 for frequency, R^2=0.0837, p-value=0.009131 for bursting, n=69 mice). **(B)** Linear regression model between average in vivo VTA DA neuron bursting and the second dimension of the PCA on activity (right) or the proportion of time spent in the T-Maze (Left) (R^2=0.0335, p-value=0.07136 for PC2, R^2=0.0508, p-value=0.0508 for T-Maze, n=69 mice). **(C)** Linear regression model between average in vivo VTA DA neuron bursting and the second dimension of the PCA on social variables (right) or time spent with another congener (Left) (R^2=0.002566, p-value=0.2823 for PC2, R^2=0.07448, p-value=0.01327 for time spent with another congener, n=69 mice). **(D)** Linear regression model between average in vivo VTA DA neuron firing frequency and the first dimension of the PCA on activity (right) or time in the T-maze per day (Left) (R^2=0.06327, p-value=0.02094 for PC2, R^2=0.07321, p-value=0.01397 for the time in the T-maze per day, n=69 mice). Red lines indicate the best fit, dashed lines the confidence interval; the estimated slope. Low value of R^2 indicates that the fit does not explain a large proportion of the variability.

A

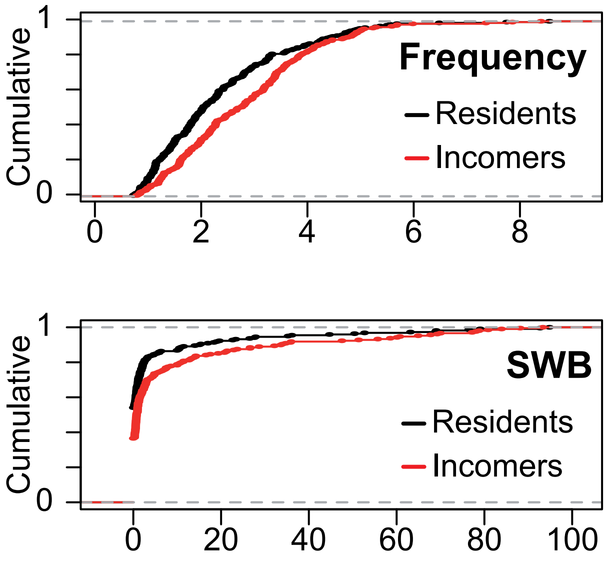

Cumulative
1
0
0
2
4
6
8
Frequency
Residents
Incomers
Cumulative
1
0
0
20
40
60
80
100
SWB
Residents
Incomers

Supp Fig 8

Supplementary Figure 8: Reversibility of the dopamine firing phenotype: (A) Cumulative distribution of cell firing frequency (top) and bursting (bottom) for residents (Black) and incomers (Red).
