# Supplementary material for: Social interactions impact on the dopaminergic system and drive individuality
Source: Nat Commun. 2018 Aug 6;9:3081. doi: 10.1038/s41467-018-05526-5 (PMC6079008; doi:10.1038/s41467-018-05526-5)
Supplement: Supplementary file 2 — Peer Review Fie [file 41467_2018_5526_MOESM2_ESM.pdf]

Reviewers' comments:

Reviewer #1 (Remarks to the Author):

Torquet et al. present an interesting perspective on the effect of a naturalistic and social environment on cognitive aspects of decision making and electrophysiological properties of VTA dopaminergic neurons. I am impressed by the experimental design and the aims of the study, and appreciate the considerable amounts of time and mice that were invested in this project. The main novelty in this work is the integration of a cognitive decision-making task within a naturalistic automatic setup, which allows dynamic and continuous analysis of mice for a period of several weeks without noticeable human interference.

However, I see no substantial novelty in the results presented, to justify publication in this journal. Moreover, this work raises some serious concerns, as detailed below.

Major comments:

1. The novelty of the experimental setup is limited at best. The integration of the cognitive task within the naturalistic environment is novel and presents several advantages (dynamic effects of social environment, the animal decides on timing etc.) that are worthy of publication. However, as far as the complex behavior, social interaction and monitoring of several mice in naturalistic environment, the setup presented is far from novel and even inferior to several distinct setups developed (and published) in recent years.

2. The novelty of the findings is also limited. While it is interesting to see different mice presenting different behavioral parameters consistently over time (=personality), there isn't too much analysis of the correlation between these behaviors. The authors conduct PCA and cluster analysis to identify distinct types of mice, but include only 4 parameters from the T maze assay (which are not even independent). Why not include all behavioral and electrophysiological parameters in the complex analysis?

3. Then, the authors use the different clusters identified and compare other parameters between the groups but the results obtained are far from impressive. First, they compare these behaviors not between the groups separated by the multi-dimensional analysis but rather by a single switch rate parameter. Second, the 3 "personalities" groups differ only by 3 presented parameters: (i) time between 2 trials (which might be expected to be related to switch rate), (ii) time in FC (which is different only for 2 of the groups), and (iii) time three or more, which actually shows no significant difference between specific groups.

4. The detection accuracy is a major concern. Within the central 1mX1m space the only detection is through the RFID antennas. This detection is not sufficient to accurately locate a single mouse with an adequate place and time resolution, and even more so for pairs or groups of mice. Moreover, the authors do not even attempt to validate the detection accuracy, which seriously dampens the reliability of their findings.

5. This poor accuracy leads to another problem, relating to identification of social behavior. According to the authors, they extract social parameters from the "simultaneous presence of a group of animals in a given space". However, there are no details regarding the parameters used to define simultaneous presence, and even if there were the resolution of RFID antennas in such dispersion is not sufficient to define social proximity. For instance, "a high rate of successive distinct RFID detections on a single antenna within short time intervals (<10 s) were indicative of social events, i.e. a group of mice passing from one compartment to another" - how can the authors even know if all the mice were passing the antenna in the same direction?
In the absence of any validation, I seriously doubt the reliability of any such social parameters.

6. The same caveats go for the definition of chasing behavior as well. In addition, the time interval for concomitant detection for the definition of chasing is described as 5s in the methods section and 10s in the legend of figure 1D. In general I'm not convinced the authors have reliably detected social behaviors, let alone chasing interactions.

7. The individual differences in decision making and in the physiological traits of the DA system are interesting, however there is no data showing that theses physiological traits are related to any social parameter (the right panel in figure 4B is very weak), or even to the duration in the naturalistic environment. These differences could have just as easily stemmed from repeated simple T-maze assays over a similar duration. A control comparison to such a group (repeated assays without naturalistic social enrichment) would have been more convincing.

Minor comments:

1. The data is presented in a very obscure manner. Some of the data is described as significant without any presentation of statistical analysis (figures 2B, S3A), while some of the graphs present n=18, others n=30, n=49 and some n=89, without any clear explanation. In other cases, such as figure 3D, it is not even properly explained what is the data presented and whether it even describes actual experimental mice.

2. The experiment presented in figure 5 is indeed interesting, however it only shows that the "individuality" parameters are not stable and depend more on environmental and social contexts than on internal factors (such as epigenetics). If anything, figure 5 suggests that switching is a result of stress.

3. There are serious gaps between the text of the manuscript and the data presented. First, the experimental setups and methodology are poorly explained and require a deep revision. For example, the 5 "sessions" are not mentioned anywhere in the methods section and it is not clear in what manner they were integrated in the experiment, whether or not all the mice underwent these 5 sessions and what was their duration.

4. The authors claim (line 68) that "social relationships govern individuality, most likely by impacting the DA system". However, there is nothing in the data, or even in the experimental design, to support this mediation claim. The authors merely analyzed DA neuronal activity following behavior. Without manipulating the DA system, their results remain correlative.

Reviewer #2 (Remarks to the Author):

In the present study, Torquet and colleague defined individual behavioral traits correlated with the dopaminergic activity. The authors designed a very interesting, and importantly, non-intrusive behavioral tracking system allowing them to parse individual behaviors across a long time scale. This innovative "Souris city" apparatus, in combination with complex analyses defined stable mice behavioral traits across challenges with limited experimenter interventions. In addition the authors performed in vivo electrophysiological recordings of ventral tegmental area (VTA) dopaminergic (DA) neurons. The authors determine that VTA DA neuron activity is correlated with the propensity of mice to adapt their behavior to a changing environment.

Overall, the study in its present form is well written, and the current data set and interpretation are accurate with the current literature. The research design is well executed and the results are extremely interesting and promising to the research field of psychiatric disorders. The current manuscript provides useful information and important insight to the role of DA neurons modulation in the emergence of individual phenotypes and the potential vulnerability for mood disorders. While I have a strong enthusiasm for the present study, the current manuscript could be enriched

by minor supplemental analyses and clarifications.

Minor comments:

1. The “Souris city” apparatus allows the authors to track daily individual behaviors across a long time period. The authors should also provide analysis beyond the current analyses of individual traits of S1-S2 transition (Figure 2), for instance they should show similar analyses for each transition: Ha-S1, S2-S3, etc… A particular interest should be made on the emergence of innate behavioral traits. Are some (and which) behavioral traits stable from Ha day1 to S1 day1?

2. The correlation between individual behavioral traits and DA neuron activity is very interesting. Mice housed in this apparatus have undergone environment enrichment, sucrose exposure and potentially present a greater motor activity than “standard housed mice”. These environmental factors have been shown to individually affect the neuronal activity. Thus the non-social and the social component of “Souris city” may differentially affect the DA neuronal activity. The authors should thus provide separated correlation analyses of DA neuron activity with social and non-social individual traits.

3. The authors should clarify the housing condition of the “standard cage” mice. Were the mice grouped housed, were the mice housed for the same time period length than for the “Souris city” mice, were they also exposed to sucrose drinking solution? According the methods section, >1Hz VTA DA neurons were recorded, but a few neurons in supplemental figure 5 appeared slower (about 0.5Hz).

4. The authors should clarify the statistical analyses performed to assess the homoscedasticity and normality of the distributions. Also, please explain how nested data (DA neurons activity) were considered.

5. Some terminology should be defined and clarified within the main text and figure legends to ease the reading process: “atypical” behavior, “Antenna 16”, “With 1” etc…

Reviewer #3 (Remarks to the Author):

This elegant study by Torquet et al. presents novel and highly relevant work and data on the behavioral tendencies of isogenic mice living in a semi-naturalistic environment. Most importantly, it relates individual differences in behavioral patters to the in vivo firing of dopaminergic neurons. The finding that behavioral tendencies and dopamine firing correlates is shifted by a modification of the social enviroment strongly supports a link between the two findings. Overall, this challenging and accomplished study is technically sound and represents a very important contribution to the understanding of the neurobiological correlates of complex behaviors and social adaptations.

I only have a few the following minor comments that could help improving the manuscript:

1. The identification of the measured traits with ‘individuality’ and ‘personality’ might appear confusing. Individuality refers to singularity of an individual. Personality traits can be ascribed to a collective or group. As referring to ‘individuality’ traits sounds counterintuitive, the authors might want to reconsider the use of this term. To what extent the reported findings relate to ‘trait’ vs ‘state’? If what is measured is individuals’ reaction to the new environment (souris-city), then, perhaps, the construct to be referred to is ‘behavioral adaptation to a novel environement’ or

similar ones.

2. L47-49: The text refers to the susceptible and resilient animals to social stress, and refers to social hierarchy analyses revealing dominant animals as being less sensitive to the effects of drugs than subordinates. Even more pertinent that the drug example, recent work showing that dominant mice are more vulnerable to social defeat stress should be mentioned in this context.

3. L66: the meaning of ‘reset’ is not clear in this sentence. Please, clarify. However, as reset normally means bringing back to default/previous state, this term does not seem to apply in this context and should be changed/corrected.

4. L106: it would be good to clarify what the text means by ‘atypical’ behaviors in this sentence.

5. L171: please, indicate to how many cohorts/groups of animals living in different souris-city groups these 5 groups of 10 mice belong to.

6. Individual differences in switching patters are modified when animals encounter a different social landscape – how to interpret this finding? What is the nature and origin of the degree of switching behavior? Could it be related to social rank?

Torquet et al, NCOMMS-18-01950

Reviewers' comments:

Reviewer #1 (Remarks to the Author):
Torquet et al. present an interesting perspective on the effect of a naturalistic and social environment on cognitive aspects of decision making and electrophysiological properties of VTA dopaminergic neurons. I am impressed by the experimental design and the aims of the study, and appreciate the considerable amounts of time and mice that were invested in this project. The main novelty in this work is the integration of a cognitive decision-making task within a naturalistic automatic setup, which allows dynamic and continuous analysis of mice for a period of several weeks without noticeable human interference.

However, I see no substantial novelty in the results presented, to justify publication in this journal. Moreover, this work raises some serious concerns, as detailed below.
We thank the reviewer for her/his very positive early comment and the fact that she/he was impressed by the design, the aim of the study and the amount of data. However, we are surprised by the lack of enthusiasm about the results and their novelty. Concerning the technical aspects and the issue of detection accuracy, we provide here below an answer and hope that these new data will convince the reviewer that detection is very reliable. As to the biological novelty and behavioral results, we re-analyzed the data, added new data and reply here below point by point to the reviewer's criticisms. We hope that these changes will clarify the novelty of our work.

Major comments:

1. The novelty of the experimental setup is limited at best. The integration of the cognitive task within the naturalistic environment is novel and presents several advantages (dynamic effects of social environment, the animal decides on timing etc.) that are worthy of publication. However, as far as the complex behavior, social interaction and monitoring of several mice in naturalistic environment, the setup presented is far from novel and even inferior to several distinct setups developed (and published) in recent years.

The integration of the cognitive task within the naturalistic environment and in a closed economy paradigm is clearly innovative and is one of the key elements that drove this work: the ability to test the cognitive functions of one animal isolated from its congeners, and to link these functions to the behavior observed in the social environment. We thank the reviewer for pointing out that such an approach is novel.

However, the reviewer regrets or suggests that the social part of our environment is far from novel and even inferior to several distinct setups developed. As of today, there are two major techniques for tracking animals:

> i) *Video recognition techniques generally based on tagged animals (e.g. coloring of the fur…).* These techniques are based on contrast and require specific, fixed experimental conditions obtained by simplifying the background or using infrared lights. They provide a high level of details in social interactions and in the position of the animal, but for a short period of time only. These techniques also require repeated manual corrections or validations of the animal identity, which prevents long-time use.

Most of these techniques are not adapted to our environment with tubes, light differences, stairs, instability in environment...

ii) *RFID-based techniques like ours.* These methods provide data over a long period of time, but with a relatively low level of detail for accurate social data. However, as we will elaborate below, the data provided by RFID detection are highly consistent, in the sense that “false localizations” are extremely rare (i.e. animals may not always be detected by the antenna, but antenna detections are always true).

For our experiments, we therefore chose the RFID strategy because it is currently the only technique that satisfies our needs.

2. The novelty of the findings is also limited. While it is interesting to see different mice presenting different behavioral parameters consistently over time (=personality), there isn’t too much analysis of the correlation between these behaviors. The authors conduct PCA and cluster analysis to identify distinct types of mice, but include only 4 parameters from the T maze assay (which are not even independent). Why not include all behavioral and electrophysiological parameters in the complex analysis?

Our objective was threefold: to establish

1) that "individuality emerges in the social environment",
2) that mice isolated from their fellows behave differently in a decision-making task
3) that these different aspects (i.e. personality, decision making) are correlated with dopaminergic activity.

We first determined whether mice behave differently in an individually-performed task, regardless of their interaction with congeners. Therefore, we took into consideration only the parameters extracted from the T maze. These parameters are initially clearly not independent. *This is why we used a PCA, a technique that transforms a set of correlated variables into linearly uncorrelated variables (the principal components), and then a clustering on this component.* This allowed us to use T-maze behaviors as an indicator of a given profile, i.e. an independent “trait” of the animal. We also want to stress that this criterion is not only quantitative, in the sense that it corresponds to a strategy.

Having demonstrated that a particular trait, here defined as the ability of the animal to switch, is a good criterion to define different subpopulations, we then used that criterion on a new pool of data (some of the experiments used in Fig 4 were not used to validate this criterion in Fig 3) and demonstrated that this “trait” is associated (Fig 4) with i) differences in the behavior of the animal in the main environment and ii) differences in the activity of the dopaminergic system. What this means is that dopamine, for instance, is not used initially as a variable to segregate subpopulation; rather, it emerges independently of the initial classification. *If we were to include all behavioral and electrophysiological parameters in the initial clustering analysis, we would not be, by definition, able to conclude that correlations exist.* Indeed, in this case, the selection process (the different animal subclasses obtained for example after a global PCA) and the analysis of some of the factors (for example the dopaminergic activity) would not be independent, and therefore there would be circularity in our reasoning. In our analysis, the differences observed in dopaminergic activity between the LS, HS and IS subgroups cannot be related to the subgroup selection process (since dopamine is not used for group selection). Same is true for all the behavioral parameters that are not directly linked with choice in the T-maze.

3. Then, the authors use the different clusters identified and compare other parameters between the groups but the results obtained are far from impressive. First, they compare these behaviors not between the groups separated by the multi-dimensional analysis but rather by a single switch rate parameter. Second, the 3 “personalities” groups differ only by 3 presented parameters: (i) time between 2 trials (which might be expected to be related to switch rate), (ii) time in FC (which is different only for 2 of the groups), and (iii) time three or more, which actually shows no significant difference between specific groups.

We agree with the reviewer that the way we presented the data may make the results look not impressive. This is partly because we have initially tried to illustrate the correlations between switching (our key distinct behavior) and very specific behaviors in the main environment (i.e. time in Food compartment, time spent alone…). We therefore reformulated the problem and categorized the set of parameters (16 variables) into three domains: distribution in spaces and activity, access to and management of the T-maze, and social aspects. The various parameters associated with each domain are now presented in Figure 4. All these parameters are a priori independent of the switching parameters. We also remind the referee that for this analysis we have not used any measure based on floor antenna detection which, although potentially informative, remains too partial (see remarks below). For the social parameters, we used the time spent with or without congeners in the sub-compartments and detections of simultaneous transitions from one compartment to another. We then performed a PCA on each group of parameters and analyzed the changes in the value of the first and second components according to whether the animal belonged to the LS, IS or HS groups. These results are now presented in Fig. 4. In addition, we now analyzed the correlations between discharge rates (Burst or frequency obtained per animal) and a certain number of behavioral parameters. These results are now added in Supp Fig. 7.

Altogether our results indicate a strong correlation between a decision-making task in which animals perform individually, the way animals organize their life in the large social environment, and the level of activity of their midbrain dopaminergic cells. Furthermore, in the last experiment, we demonstrate that a change in social properties, or otherwise-stated a change in the relation between congeners, spreads to others domains and is associated with a modification in the dopamine system and in the decision-making task. These results are clearly novel and highlight the importance of the social context on individuations.

4. The detection accuracy is a major concern. Within the central 1mX1m space the only detection is through the RFID antennas. This detection is not sufficient to accurately locate a single mouse with an adequate place and time resolution, and even more so for pairs or groups of mice. Moreover, the authors do not even attempt to validate the detection accuracy, which seriously dampens the reliability of their findings.

Detection accuracy is indeed an important point. The social cage is divided into four sub-compartments (see Supp Fig. 1 et 2): NC, which contains a nest, FC where mice have free and uncontrolled access to food, CC and St to get access to the gate. NC, FC and CC are located in a 1m x 1m square. We used the RFID technique to identify and locate each mouse. Detection is based on three independent systems:

- The compartments are separated by walls. The transition from one compartment to the other is made through tubes equipped with two circular antennas. Therefore, each transition from one compartment to the other was associated with a detection of the animal by the two consecutive antennas, thus allowing to readout the direction. These antennas are controlled by the Controller software (TSE systems).

- One last antenna system is on the gate itself. The gate opens only upon detection of an animal. For this circular antenna, there is therefore no problem of detection accuracy. The gate and its antenna are controlled by the Controller software, like for the tube antennas.
- RFID antennas controlled by the TraffiCage software (TSE systems) are located below the floor of the entire social environment

The question of the validity of detection is therefore two-fold: First what is the reliability of localization in a given compartment and, second, what is the reliability of detection at the level of a given antenna. We now show new data demonstrating the reliability of the detection in both conditions (Supp Fig. 2). When an animal transits from one compartment to another, it is detected in 100% of the cases, *thus demonstrating a very high reliability of detection of an individual in a given sub-compartment (i.e. FC, NC...).* Regarding reliability of detection at the level of floor antennas, our data indicate that animals are detected "only" 75% of the time when their trajectory crosses the antenna, and that there are very few (if any) false detections. Non-detections can be due to i) the animal bypassing the detection area of a given antenna ii) the animal not being detected due to its high speed iii) multiple animals being present on the same antenna (in case two mice are simultaneously present on a given antenna, only one is detected). Finally, in order to validate the very high reliability of the detection of an individual in a given compartment, we cross-checked detection by the two systems (tube and floor antennas). Overall, we found that localization by both systems in a given compartment is consistent in more than 99.96% of the cases. Using trajectories and sequences of compartment visits, we further validate our system by showing the total absence of forbidden sequences (e.g. from NC to FC, two unconnected compartments).

We discuss the consequences of this detection process below (see questions 5 and 6), but we here want to stress that floor antenna information is only used for few parameters in this study and only for the quantification of the reliability of behaviors across sessions. This is notably the case for the chasing behavior parameters that were obtained using all antennas (Floor and Tube).

5. This poor accuracy leads to another problem, relating to identification of social behavior. According to the authors, they extract social parameters from the "simultaneous presence of a group of animals in a given space". However, there are no details regarding the parameters used to define simultaneous presence, and even if there were the resolution of RFID antennas in such dispersion is not sufficient to define social proximity. For instance, "a high rate of successive distinct RFID detections on a single antenna within short time intervals (<10 s) were indicative of social events, i.e. a group of mice passing from one compartment to another" - how can the authors even know if all the mice were passing the antenna in the same direction?
In the absence of any validation, I seriously doubt the reliability of any such social parameters.

We understand the reviewers' concerns, but we think there is here still a confusion that needs to be clarified. Indeed, we derived a first series of social parameters from animal position in a given sub-compartment and quantified the "simultaneous presence of a group of animals in a given space". Here "space" refers to the sub-compartments and not to the floor antennas. There is therefore no doubt about the validity of this social parameter. A second type of social parameters were derived from the information about the transitions from one compartment to another. These transitions are detected by two separated tube antennas. High rate of successive RFID detections on tube antennas within short time intervals (<10 s) thus indicates sequential transitions from one compartment to another. 10 s is here an observation (see Fig.

1D). Again, there is no use of floor antenna parameters and all tube detections are highly reliable. Finally, we introduce another social parameter ("Follower / leader" Fig. 1E), which is based on floor antenna detections (see question 6).
To avoid confusion, we now have rephrased sentences and decomposed panels 1D and 1E.

6. The same caveats go for the definition of chasing behavior as well. In addition, the time interval for concomitant detection for the definition of chasing is described as 5s in the methods section and 10s in the legend of figure 1D. In general I'm not convinced the authors have reliably detected social behaviors, let alone chasing interactions.

As explained in the methods, *"Chasing episodes were defined by concomitant (i.e. within a 5s window) detections of the same two mice on at least two consecutive antennas. Antennas were considered consecutive if the first mouse from a concomitant detection on one antenna was detected within a 30s window on another antenna"*. 10 s in the legend figure refers to the current Fig. 1D that shows transition from one compartment to another using tube antenna. We are sorry for the misunderstanding and, as stated above, we have now isolated the chasing description (Fig. 1E) from the coincident transition detections (Fig. 1D).

Tracking behavior is the only parameter analyzed in this study that derives from floor antenna detections. As previously explained, the floor antenna system is not perfect and may fail to capture mice crossover (see above). However, analysis of these data shows a strong correlation between the number of chasing episodes from one session to the next, indicating a consistency in the behavior of individuals. This measure allows us to highlight this consistency but does not provide an explanation on the origin of this difference. We took care to not over-interpret these results, and did not use this parameter in the subsequent analysis of the current version of the paper (Fig. 3,4 and 5). This is now clearly stated in the manuscript (page 5 and methods).

7. The individual differences in decision making and in the physiological traits of the DA system are interesting, however there is no data showing that theses physiological traits are related to any social parameter (the right panel in figure 4B is very weak), or even to the duration in the naturalistic environment. These differences could have just as easily stemmed from repeated simple T-maze assays over a similar duration. A control comparison to such a group (repeated assays without naturalistic social enrichment) would have been more convincing.

Here we must make a clear distinction between a correlation between dopamine and a social parameter, and the manipulation of a "social context". Once again, the first part of the work allows correlations to be established and shows that a profile of choice in a task performed in isolation from congeners correlates with differences both in dopaminergic activity and in the behaviors in the social compartment. These correlations are not weak, the three groups analysis (Fig. 4B) showed group effect ($p<0.01$).

The second question is whether these differences can result from simple and repeated tests over a similar period of time. Do we need such a complex system to demonstrate differences in individual behaviors? Perhaps not, but as we have already mentioned, we are not only seeking to demonstrate that differences exist within sub-populations (such as resilience versus sensitivity or other examples in the literature), we are also seeking to understand how these differences propagate or emerge from differences in general individual behaviors. Finally, we aimed to dissect the mechanisms underlying these differences. By primarily manipulating the

social context, our method allows to show how behavioral (switching) and electrophysiological parameters adapt, regardless of the past history or of any particular genetic underpinning. This paper deals with individual behaviors and their emergence. These questions cannot be addressed using a simple test, without controlling the individual's history or group life.

Minor comments:

1. The data is presented in a very obscure manner. Some of the data is described as significant without any presentation of statistical analysis (figures 2B, S3A), while some of the graphs present n=18, others n=30, n=49 and some n=89, without any clear explanation. In other cases, such as figure 3D, it is not even properly explained what is the data presented and whether it even describes actual experimental mice.

The reviewer is right, sorry about the confusion. We now provide the reader with a table explaining the actual experiments that are used in a given figure.

2. The experiment presented in figure 5 is indeed interesting, however it only shows that the “individuality” parameters are not stable and depend more on environmental and social contexts than on internal factors (such as epigenetics). If anything, figure 5 suggests that switching is a result of stress.

We thank the reviewer for finally recognizing a demonstration that individual parameters are not stable and depend more on environmental and social contexts than on internal factors (such as epigenetics). However, we will point out that this notion of stability is relative. As long as the (social) environment is stable, the parameters of individuality are stable (this is the first part of the paper). On the other hand, if we modify a condition, in our case solely the social aspect, the system readjusts and is not stuck by genetics. Figure 5 demonstrates this specific point. Stress may indeed be a factor involved in changes in behavior, but in the absence of an easy and reliable way of measuring stress in animals (which of the incomers or residents is under more stress?), we think it is more appropriate to analyze our results in terms of changes in social context, which is a controllable variable. Furthermore, we think stress has a negligible effect. Indeed, if we suppose that the change in social environment stresses animals, then the overall distribution of switch rate would be different before and after mixing, which is not what we observed. In addition, the idea that stress may affect differently incomers versus residents is hard to reconcile with the fact that LS residents modified their switch rate after the social challenge.

3. There are serious gaps between the text of the manuscript and the data presented. First, the experimental setups and methodology are poorly explained and require a deep revision. For example, the 5 “sessions” are not mentioned anywhere in the methods section and it is not clear in what manner they were integrated in the experiment, whether or not all the mice underwent these 5 sessions and what was their duration.

We thank the reviewer and took note of her/his remarks. We have now an entire section in the supplementary material that describes the setup, experiments, and validation of some measurements. We also better explain the different sessions (page 6 and 7). We hope that all these data will provide a better understanding of the tasks.

4. The authors claim (line 68) that “social relationships govern individuality, most likely by

impacting the DA system". However, there is nothing in the data, or even in the experimental design, to support this mediation claim. The authors merely analyzed DA neuronal activity following behavior. Without manipulating the DA system, their results remain correlative.

We rephrased the sentence. As explained before, we manipulated the social relationship (at a global level) and analyzed the consequences on decision and at the level of the VTA. This allows to conclude that social relationships impact on both behavior and dopaminergic system. The new sentence is now:
Social relationships impact individuality, probably by regulating the activity of the DAergic system.

Reviewer #2 (Remarks to the Author):

In the present study, Torquet and colleague defined individual behavioral traits correlated with the dopaminergic activity. The authors designed a very interesting, and importantly, non-intrusive behavioral tracking system allowing them to parse individual behaviors across a long time scale. This innovative “Souris city” apparatus, in combination with complex analyses defined stable mice behavioral traits across challenges with limited experimenter interventions. In addition the authors performed in vivo electrophysiological recordings of ventral tegmental area (VTA) dopaminergic (DA) neurons. The authors determine that VTA DA neuron activity is correlated with the propensity of mice to adapt their behavior to a changing environment.

Overall, the study in its present form is well written, and the current data set and interpretation are accurate with the current literature. The research design is well executed and the results are extremely interesting and promising to the research field of psychiatric disorders. The current manuscript provides useful information and important insight to the role of DA neurons modulation in the emergence of individual phenotypes and the potential vulnerability for mood disorders. While I have a strong enthusiasm for the present study, the current manuscript could be enriched by minor supplemental analyses and clarifications.

We thank the reviewer for his/her very positive comment. We hope that this new version, which integrates her/his comments, will satisfy her/him even further.

Minor comments:

1. The “Souris city” apparatus allows the authors to track daily individual behaviors across a long time period. The authors should also provide analysis beyond the current analyses of individual traits of S1-S2 transition (Figure 2), for instance they should show similar analyses for each transition: Ha-S1, S2-S3, etc… A particular interest should be made on the emergence of innate behavioral traits. Are some (and which) behavioral traits stable from Ha day1 to S1 day1?
We agree with the reviewer that the definition of innate behavioral traits is interesting. The reviewer question is mainly linked to the difference between trait and state (see also reviewer three). “Prototypical traits are stable, long lasting and internally caused. Prototypical states are temporary, brief, and caused by external circumstances.” Thus, despite often intertwined, traits and states refer to the idea of habitual versus transitory patterns of behavior, respectively. Innate behaviors are linked to the notion of trait; in contrast the notion of emergence of traits or states is much more complicated. In our experiments, the measured variables (time spent in a given sub-compartment…) may relate to both state or trait, but we insist on the fact that we aim to make distinction between different individualities. In the notion of individuality, what remains stable is not the measured trait or state, but the difference among individuals (i.e. if mouse # i explores more than mouse # j, this remains the case day after day).

2. The correlation between individual behavioral traits and DA neuron activity is very interesting. Mice housed in this apparatus have undergone environment enrichment, sucrose exposure and potentially present a greater motor activity than “standard housed mice”. These environmental factors have been shown to individually affect the neuronal activity. Thus the non-social and the social component of “Souris city” may differentially affect the DA

neuronal activity. The authors should thus provide separated correlation analyses of DA neuron activity with social and non-social individual traits.

The reviewer is right when she/he says that locomotor activity, sucrose or enriched environments can modulate dopaminergic activity. Sucrose exposure is a factor that can easily be tested. We now analyzed the effect of sucrose exposure on VTA DA cell activity (see question 3) and report a moderate increase in bursting activity. This increase contrasts with the decreased DA cell activity observed in HS mice.

Non-social and social components of "Souris City" may differentially affect DA neuronal activity, but they also affect each other, since they are not independent. However, in order to clarify our findings, we have now categorized the set of parameters into three domains: distribution in spaces and activity, access to and management of the T-maze, and aspects of group behavior. The various parameters in each domain are presented as additional material. All these parameters are a priori independent of the choice of the animal in the T-maze, and for sure independent of the parameters used to characterize decision making. We then performed a PCA on each group of parameters and analyzed the variations in the value of the first and second components according to whether the animal belongs to the LS, IS or HS group (Fig. 4A,B). These novel analyses reveal strong correlations among categories of parameters and switching. In addition, we now analyzed the correlations between discharge rates (Burst or frequency obtained per animal) and a certain number of behavioral parameters. These results are now added in Supp Fig. 7 and confirm some correlations between neuron discharge parameters and the three main classes of parameters. We also want to emphasize that, clearly, dopaminergic activity can be influenced by a complex network of environmental conditions. What we show here is that "recent social history" can influence individual traits and the dopamine system.

3. The authors should clarify the housing condition of the "standard cage" mice. Were the mice grouped housed, were the mice housed for the same time period length than for the "Souris city" mice, were they also exposed to sucrose drinking solution? According the methods section, >1Hz VTA DA neurons were recorded, but a few neurons in supplemental figure 5 appeared slower (about 0.5Hz).

For the control group, mice are received at the age of 8 weeks, bred in cages of 5 for 2-4 weeks with water, and then dopaminergic activity is recorded. The values obtained are compatible with those obtained in the laboratories for such a "standard housed" situation and for animals of that age. The reviewer is right when she/he suggests that a comparison with mice exposed to sucrose can be useful. We thus exposed animals in their home cage to sucrose instead of water and analyzed the effect of sucrose exposure on VTA DA cell activity. Our analyses show that there is no modification of VTA DA cell frequency when compared to the control group, but a small increase in bursting activity. The overall difference observed in Souris-City is thus certainly not simply due to sucrose exposure, but rather to a complex interaction between environment, social exposure and possibly sucrose. We now detailed (page 15) the housing condition.

Concerning VTA DA neuron recordings, cells were identified on the basis of their electrophysiological activity as indicated in the method section. In our experiments, we also labeled some neurons with neurobiotin and used immunohistochemistry (TH) to identified them as dopaminergic. The few cells recorded with a firing rate below 1 Hz were identified as dopaminergic using immunochemistry. This is now added in the methods section (page 21)

4. The authors should clarify the statistical analyses performed to assess the homoscedasticity and normality of the distributions. Also, please explain how nested data (DA neurons activity) were considered.

Statistic are now precisely defined in the methods section. Overall, when data were normally distributed (Shapiro-Wilk Normality Test), we performed a one-way anova (generally with three groups) followed by post-hoc test (Tukey's range test i.e TukeyHSD test). When data were not normally distributed or variance not equal, we performed the Kruskal–Wallis test for testing whether multiple samples originate from the same distribution. This test was followed by a post-hoc test, i.e. Wilcoxon rank test with Holm's sequential Bonferroni p-value correction. This is now clearly stated in the supplementary material (page 21).
For each recorded neuron, the mean firing frequency and mean bursting activity were evaluated on a basis of a least 10 minutes of recordings. These mean values were used to characterize each neuron. Animal firing activity was estimated by pooling the activity from each neuron recorded in a given animal and estimated by a mean value. This is now clearly stated in the methods section (page 22).

5. Some terminology should be defined and clarified within the main text and figure legends to ease the reading process: “atypical” behavior, “Antenna 16”, “With 1” etc…

We thank the referee. We agree that “Antenna 16” parameter may sound strange. We now clearly make differences between parameters used to estimate stability of behavior across sessions (Fig. 2) and parameters used to quantify behavior (Fig. 4A). The first one also integrates a series of raw data such as the number of detection on a specific antenna (e.g. “Antenna 16”), or the detection of sequence of simultaneous detections (Fig. 2G, Supp Fig. 4 and methods). We also changed “atypical” behavior by “atypical profiles”. For instance, mice #13 diverges from the group.

Reviewer #3 (Remarks to the Author):

This elegant study by Torquet et al. presents novel and highly relevant work and data on the behavioral tendencies of isogenic mice living in a semi-naturalistic environment. Most importantly, it relates individual differences in behavioral patters to the in vivo firing of dopaminergic neurons. The finding that behavioral tendencies and dopamine firing correlates is shifted by a modification of the social environment strongly supports a link between the two findings. Overall, this challenging and accomplished study is technically sound and represents a very important contribution to the understanding of the neurobiological correlates of complex behaviors and social adaptations.

We thank the reviewer for his/her very positive comment. We hope that this new version that integrates her/his comments will satisfy her/him just as much.

I only have a few the following minor comments that could help improving the manuscript:

1. The identification of the measured traits with ‘individuality’ and ‘personality’ might appear confusing. Individuality refers to singularity of an individual. Personality traits can be ascribed to a collective or group. As referring to ‘individuality’ traits sounds counterintuitive, the authors might want to reconsider the use of this term. To what extent the reported findings relate to ‘trait’ vs ‘state’? If what is measured is individuals’ reaction to the new environment (souris-city), then, perhaps, the construct to be referred to is ‘behavioral adaptation to a novel environement’ or similar ones.

Concerning the confusion between personality and individuality: We defined individuality “as differences that remain stable over time and contexts for a series of behavioral traits expressed among individuals of the same species”, a definition shared by numerous authors. It thus refers to singularity of an individual. As pointed out by the reviewer, individuality and personality are not synonymous. In order to avoid any confusion, we took care to use “individuality” in the appropriate context and avoid the use of “personality”. We also removed the use of “individuality trait” and minimized the use of “individual traits”.

Second, concerning “State” an “trait”. “Prototypical traits are stable, long lasting and internally caused. Prototypical states are temporary, brief, and caused by external circumstances.” Thus, despite often intertwined, trait and state refer to the idea of habitual and transient patterns of behavior, respectively. In our experiments, the measured variables (time spent in a given sub-compartment…) may indeed relate to both state or trait, but what we are mostly interested in are individualities. In the notion of individuality, what remains stable is not the measured traits or states, but the differences among individuals (e.g. if mouse # i explores more than mouse # j, this remains true day after day).

Finally, we do not just measure “individual’ reactions to the new environment, rather we aim at evaluating differences between individuals, by testing how the social environment can reshape traits. All traits depend on both genetic and environmental factors.

We thus kept the notion of trait in the manuscript but now discuss the referee’s point (page 11).

2. L47-49: The text refers to the susceptible and resilient animals to social stress, and refers to social hierarchy analyses revealing dominant animals as being less sensitive to the effects of

drugs than subordinates. Even more pertinent that the drug example, recent work showing that dominant mice are more vulnerable to social defeat stress should be mentioned in this context.
Reviewer is right. We now mentioned this work.

3. L66: the meaning of ‘reset’ is not clear in this sentence. Please, clarify. However, as reset normally means bringing back to default/previous state, this term does not seem to apply in this context and should be changed/corrected.
We agree with the reviewer and we changed it for ‘modify’ which is more appropriate.

4. L106: it would be good to clarify what the text means by ‘atypical’ behaviors in this sentence.
Indeed, what we meant is “atypical profiles” and not behaviors. For instance, mice #13 diverges from the group. We modified the sentence.

5. L171: please, indicate to how many cohorts/groups of animals living in different souris-city groups these 5 groups of 10 mice belong to.
We thank the referee for this comment. We now added a tab (Materials and Methods, page 20) explaining in details the number of mice in the different groups.

6. Individual differences in switching patters are modified when animals encounter a different social landscape – how to interpret this finding? What is the nature and origin of the degree of switching behavior? Could it be related to social rank?
These are indeed outstanding questions, for which we don’t have a definitive answer unfortunately. Social ranking may have a strong impact of switching patterns, but measuring it isn’t trivial at all within Souris City. Currently we can only speculate on the origin of switching behavior (see discussion) and in the current state we prefer not to over-interpret our data.

REVIEWERS' COMMENTS:

Reviewer #1 (Remarks to the Author):

The authors have made considerable revisions to their manuscript and answered most of my previous reservations. The addition of the detection validations is an important supplement to the experimental design, and I agree that the majority of results displayed cannot be attributed to any possible detection errors.
I do have two more suggestions for the authors before the final publication:

1. As the authors did not present any validation for accurate detection of complex social interactions (e.g. chasing events), and since such parameters were not included in the PCA of social parameters in Figure 4B anyway; I would refrain from claiming the system can reliably detect such complex social parameters.

2. In response to my final comment in the previous version, the authors changed: “social relationships govern individuality, most likely by impacting the DA system” to: "Social relationships impact individuality, probably by regulating the activity of the DAergic system". This is basically the same. As the authors did not perform any manipulation on the DAergic system, they can only suggest it as a possible mediator (not a probable one).

Reviewer #2 (Remarks to the Author):

Upon review, N. Torquet and collaborators have adequately revised the manuscript. They have included new control experiments and extended the discussion to rule out alternative hypotheses. Based on the reviewer comments, the authors have also reshaped the manuscript, improving the interpretation of the results. Finally, the authors have addressed all the comments point by point. All the provided modifications strongly improve the clarity of their study and increase the significance of their research. The research and experimental design on which this manuscript is based is intrinsically interesting. I have no further comments.

Reviewer #3 (Remarks to the Author):

The authors have addressed my previous questions satisfactorily.
However, please check for typos and grammar; e.g.:

- 'don't' should be corrected by 'did not' in the following sentence "In one 447 experiment, one mice that don’t drink was rapidly exclude".

- page 12, first line: some words seem to be missing "of these differences when faced with changes" - it is not clear what type of changes the sentence relates to.

REVIEWERS' COMMENTS:

Reviewer #1 (Remarks to the Author):

The authors have made considerable revisions to their manuscript and answered most of my previous reservations. The addition of the detection validations is an important supplement to the experimental design, and I agree that the majority of results displayed cannot be attributed to any possible detection errors.
I do have two more suggestions for the authors before the final publication:
We thank the referee for her/his comments.

1. As the authors did not present any validation for accurate detection of complex social interactions (e.g. chasing events), and since such parameters were not included in the PCA of social parameters in Figure 4B anyway; I would refrain from claiming the system can reliably detect such complex social parameters.
We agree with the referee. We now added two sentences that clearly state that such parameters were not used in this system.
P9 Line 209-211: To avoid potential pitfalls associated with missed detections, variables derived from floor antennas (such as chasing episode) were not used here.
P18: Lines 443-446 in the method section: "Because the floor antenna system is fully accurate and may fail to capture mice crossover (see Supplementary Fig 2), measures derived from floor detections were only used to highlight animal consistency (Fig 2) but were not used in subsequent analyses (i.e. Fig 3, 4 and 5).".
Furthermore, we added a sentence (P5) to explicitly mention the limits of the detection accuracy in the results section, and to refer to the new supplementary figure 2: "These detections were overall highly reliable (see limits in Supplementary Fig.2), leading to an unambiguous global representation of mouse distribution".

2. In response to my final comment in the previous version, the authors changed: "social relationships govern individuality, most likely by impacting the DA system" to: "Social relationships impact individuality, probably by regulating the activity of the DAergic system". This is basically the same. As the authors did not perform any manipulation on the DAergic system, they can only suggest it as a possible mediator (not a probable one).
We agree with the referee and we tuned down our claim by changing "probably" to "possibly".

Reviewer #2 (Remarks to the Author):

Upon review, N. Torquet and collaborators have adequately revised the manuscript. They have included new control experiments and extended the discussion to rule out alternative hypotheses. Based on the reviewer comments, the authors have also reshaped the manuscript, improving the interpretation of the results. Finally, the authors have addressed all the comments point by point. All the provided modifications strongly improve the clarity of their study and increase the significance of their research. The research and experimental design on which this manuscript is based is intrinsically interesting. I have no further comments.

We thank the referee for her/his comments.

Reviewer #3 (Remarks to the Author):

The authors have addressed my previous questions satisfactorily.
We thank the referee for her/his comments.

However, please check for typos and grammar; e.g.:

- 'don't' should be corrected by 'did not' in the following sentence "In one 447 experiment, one mice that don't drink was rapidly exclude".
We modified the sentence accordingly and verified the entire manuscript for typos and grammar.

- page 12, first line: some words seem to be missing "of these differences when faced with changes" - it is not clear what type of changes the sentence relates to.
Indeed. It has been changed to: " of these differences when animals were faced with environmental changes".
